# Tumor-Secreted ADAMTSL4 Activates Latent TGFβ1 to Drive Cancer Cachexia

**DOI:** 10.1158/2159-8290.CD-26-0045

**Published:** 2026-09-01

**Authors:** Juliano Machado, Vignesh Karthikaisamy, Hermine Mohr, Doris Kaltenecker, Pia Benedikt, Pauline Morigny, Amit Mhamane, Julia Geppert, Amy Rose Fumo, Kerstin Haase, Estefania Simoes, Joanna D.C.C. Lima, Anastasia Georgiadi, Achim Krüger, José Pinhata Otoch, Marc E. Martignoni, Vickie E. Baracos, Mariam Jamal-Hanjani, Marilia C.L. Seelaender, Olga Prokopchuk, Julia Szendrödi, Maria Rohm, Stephan Herzig, Mauricio Berriel Diaz

**Affiliations:** 1 Institute for Diabetes and Cancer (IDC), Helmholtz Munich, German Research Center for Environmental Health, Neuherberg, Germany.; 2 Joint Heidelberg-IDC Translational Diabetes Program, Heidelberg University Hospital, Heidelberg, Germany.; 3 German Center for Diabetes Research (DZD), Neuherberg, Germany.; 4 International Helmholtz Research School for Diabetes, Helmholtz Munich, Neuherberg, Germany.; 5 Cancer Research UK Lung Cancer Centre of Excellence, University College London Cancer Institute, London, United Kingdom.; 6 Cancer Metastasis Lab, University College London Cancer Institute, London, United Kingdom.; 7 Department of Oncology, University College London Hospitals, London, United Kingdom.; 8 Cancer Metabolism Research Group, LIM 26 HCFMUSP and Department of Surgery, Faculdade de Medicina, University of São Paulo, São Paulo, Brazil.; 9 Institute of Experimental Oncology and Therapy Research, Technical University of Munich, School of Medicine, Munich, Germany.; 10 University Hospital, University of São Paulo, São Paulo, Brazil.; 11 Department of Surgery, Klinikum rechts der Isar, School of Medicine, Technical University of Munich, Munich, Germany.; 12 Division of Palliative Care Medicine, Department of Oncology, Cross Cancer Institute, University of Alberta, Edmonton, Canada.; 13 Chair Molecular Metabolic Control, Technical University of Munich, Munich, Germany.

## Abstract

Cancer cachexia is a devastating wasting syndrome with no approved therapies. In this study, we identify the tumor-derived glycoprotein ADAMTSL4 as a circulating factor associated with body weight loss in preclinical cachexia models and patients with colorectal and lung cancers. In mice, *Adamtsl4* overexpression converted non–cachexia-inducing tumors into cachexia-inducing tumors, whereas its deletion in cachexia-inducing tumors spared fat and muscle, blunted muscle atrophy signatures, and reduced cachexia severity. ADAMTSL4 engages the latency-associated peptide (LAP) of TGFβ1, promoting local activation of TGFβ1 at muscle cell membranes. Genetic blockade of proTGFβ1 or pharmacologic inhibition of TGFβ signaling reduced ADAMTSL4-dependent wasting in adipocytes and muscle cells. Suppression of tumor-derived ADAMTSL4 attenuated skeletal muscle fibrosis in mice. Together, the association between increased circulating ADAMTSL4 levels and TGFβ–driven muscle atrophy and fibrosis gene signatures in patients with cachectic cancer identifies ADAMTSL4 as an upstream regulator of TGFβ1 and a potential therapeutic target in cancer cachexia.

**Significance:**

Cancer cachexia lacks effective therapies and remains a major cause of cancer-related morbidity and mortality. We identify tumor-derived ADAMTSL4 as an upstream regulator of latent TGFβ activation via LAP engagement that promotes multiorgan wasting and fibrosis-related remodeling. Targeting ADAMTSL4 may provide a selective therapeutic strategy without systemic TGFβ pathway blockade.

*See related commentary by Liu and Janowitz, p. 1964*

## Teaser

Tumor-secreted ADAMTSL4 activates latent TGFβ1 at muscle cell surfaces, promoting muscle atrophy and fibrosis while also driving adipose tissue wasting, and represents a selective therapeutic target for cancer cachexia.

## Introduction

Cancer cachexia is a multifactorial wasting disorder characterized by involuntary loss of skeletal muscle mass, with or without loss of fat mass, that cannot be fully reversed by conventional nutritional support (1). It affects approximately 50% of patients with cancer and significantly contributes to cancer-related morbidity and mortality (2). Cancer cachexia impairs quality of life, reduces tolerance to anticancer therapies, and imposes a substantial burden on patients, caregivers, and healthcare systems, underscoring the urgent need for effective therapeutic strategies. Although cancer cachexia arises from complex interactions between tumor- and host-derived factors across multiple organs and tissues, tumor-secreted mediators play a central role in initiating and sustaining tissue wasting (2). However, the molecular mechanisms linking tumor-derived signals to systemic catabolism remain incompletely understood. Several tumor-derived mediators, notably proinflammatory cytokines such as interleukin-6 (IL6) and leukemia inhibitory factor (LIF), as well as TGFβ family members, including GDF15 and activin A, have been implicated in cachexia (3–6).

Tumor-secreted factors, including IL6, PTHrP, activin A, and TGFβ, promote cachexia through direct effects on skeletal muscle and adipose tissue (6–9), and some additionally act via the central nervous system. For example, GDF15 signals through the area postrema (AP) and nucleus of the solitary tract (NST) to induce anorexia and weight loss (10, 11), whereas IL6 can promote apathy-like behaviors through AP–parabrachial circuits (12). Although inhibition of IL6 shows partial benefit in preclinical models (13), these approaches have not translated into robust clinical efficacy. In contrast, targeting GDF15 with the monoclonal antibody ponsegromab demonstrated promising effects in early-phase clinical trials, including weight gain and symptom improvement in patients with elevated circulating GDF15 (14–16). Nonetheless, not all cachectic patients exhibit elevated GDF15, and some studies found no association between tumor GDF15 expression and cachexia prevalence in advanced non–small cell lung cancer (NSCLC; ref. 17). These findings underscore the biological heterogeneity of cancer cachexia and highlight the need to identify additional tumor-derived mediators to develop more personalized and effective therapies, particularly given the lack of FDA-approved drugs.

Aiming to identify novel tumor-derived mediators of cancer cachexia, we performed integrative pan-cancer transcriptomic analyses of the ADAMTS-like family, which identified ADAMTSL4 as the most prominent member associated with poor overall survival (OS) and tumor microenvironment remodeling across multiple solid tumor types (18). Given that cancer cachexia is strongly associated with adverse survival outcomes, particularly in colorectal and lung cancers (19), we hypothesized that extracellular matrix (ECM)-associated factors linked to aggressive tumor biology may represent candidate mediators of systemic metabolic decline. Supporting this, an independent cell-selective secretome study using pancreatic cancer cells cocultured with macrophages identified ADAMTSL4 among the top secreted ECM regulators detected in both tumor and macrophage compartments, consistent with a role in tumor–stroma communication (20). Likewise, secretome profiling of monocytes exposed to colorectal cancer cells identified ADAMTSL4 as a candidate factor associated with unfavorable prognostic outcomes in patients with colorectal cancer (21). Together, these independent observations suggest that tumor-derived ADAMTSL4 may represent a previously unrecognized extracellular mediator linked to aggressive cancer phenotypes, including cancer cachexia. Using genetic gain- and loss-of-function approaches in tumor allografts, together with analyses of circulating ADAMTSL4 and muscle tissue, we aimed to define how this factor influences host metabolism. We further explored the mechanisms by which ADAMTSL4 influences muscle and adipose tissue homeostasis, positioning ADAMTSL4 as a candidate upstream regulator of tissue-level TGFβ activation and a potential therapeutic target in cancer cachexia.

## Results

### Tumor-Secreted ADAMTSL4 Is Elevated in Preclinical Models of Cancer cachexia

Cachexia is an independent predictor of poor OS in patients with colorectal, pancreatic, and lung cancers (19, 22). Given the association of ADAMTSL4 with poor prognosis across multiple solid tumor types (19, 22), we investigated whether elevated circulating ADAMTSL4 levels are linked to cancer cachexia *in vivo*. To this end, we assessed several preclinical mouse models of cancer cachexia representing different tumor types and varying degrees of body weight loss.

**Figure 1.**
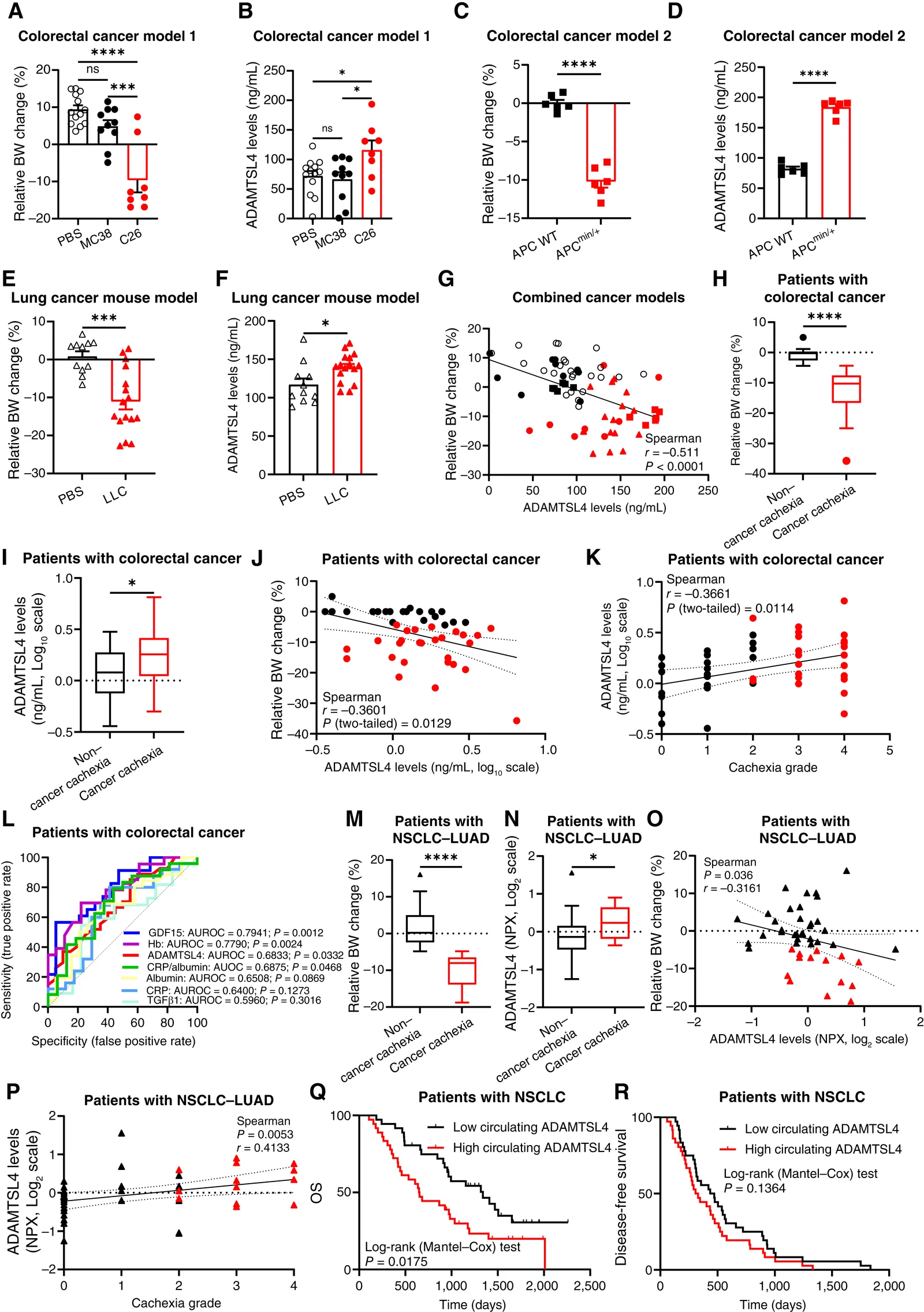
Circulating ADAMTSL4 levels are elevated in cancer cachexia. **A** and **B,** Relative body weight (BW) change (% of initial BW; **A**) and plasma ADAMTSL4 protein levels measured by ELISA (**B**) in cachectic C26 tumor-bearing mice (*n* = 8), noncachectic MC38 tumor-bearing mice (*n* = 10), and PBS-injected controls (*n* = 13). **C** and **D,** Relative BW change (**C**) and plasma ADAMTSL4 levels (**D**) in cachectic *Apc*^Min/+^ mice (*n* = 6) vs. wild-type (WT) littermates (*n* = 6). **E** and **F,** Relative BW change (**E**) and plasma ADAMTSL4 levels (**F**) in cachectic LLC tumor-bearing mice (*n* = 16) and PBS-injected controls (*n* = 11). **G,** Spearman correlation between plasma ADAMTSL4 levels and relative BW change across all mouse models (*n* = 70 XY pairs; pooled from **A–F**). **H** and **I,** Relative BW change (**H**) and circulating ADAMTSL4 levels (**I**; ELISA, log_10_ scale) in noncachectic (non–cancer cachexia, *n* = 20) and cachectic (cancer cachexia, *n* = 27) patients with colorectal cancer. **J** and **K,** Spearman correlations in patients with colorectal cancer between circulating ADAMTSL4 levels and relative BW change (**J**; *n* = 47 XY pairs) or cachexia grade (**K**; *n* = 47 XY pairs). **L,** ROC analysis comparing circulating ADAMTSL4 with established cachexia-associated biomarkers in patients with colorectal cancer. **M** and **N**, Relative BW change (**M**) and circulating ADAMTSL4 protein levels (**N**) in noncachectic (*n* = 30) and cachectic (*n* = 14) patients with LUAD from the TRACERx cohort. ADAMTSL4 was quantified by Olink proximity extension assay and is reported as NPX (log_2_-scaled relative abundance units). **O** and **P,** Spearman correlations between ADAMTSL4 NPX values and relative BW change (**O**; *n* = 44 XY pairs) or cachexia grade (**P**; *n* = 44 XY pairs) in patients with LUAD. **Q,** Kaplan–Meier analysis of OS in patients with NSCLC from the TRACERx cohort stratified by high vs. low circulating ADAMTSL4 (median NPX as cutoff). **R,** Kaplan–Meier analysis of disease-free survival in patients with NSCLC from the TRACERx cohort stratified by high vs. low circulating ADAMTSL4 (median NPX as cutoff). Data are presented as the mean ± SEM for mouse experiments (**A–F**). Relative BW change in patient cohorts (**H** and **M**) is shown with individual data points and mean ± SEM. For human biomarker group comparisons (**I** and **N**), data are shown as box-and-whisker plots with individual data points overlaid. Boxes indicate the interquartile range, center lines indicate the median, and whiskers indicate Tukey whiskers (1.5 × IQR). ELISA-based ADAMTSL4 concentrations in patients with colorectal cancer (**I**) are shown on a log_10_-transformed scale, whereas circulating ADAMTSL4 levels in patients with LUAD (**N**) are presented as NPX values (log_2_ scale). Due to differences in measurement platforms, values were analyzed within each cohort and were not directly compared across datasets. *Statistical analysis*: one-way ANOVA with Tukey multiple-comparisons test (**A** and **B**), two-tailed unpaired *t* test (**C–F**), and Spearman correlation (**G**). Human data were systematically evaluated for normality and outliers (ROUT, Q = 1%). For ELISA-based comparisons in colorectal cancer (**I**), distributions were right-skewed; therefore, two-tailed Mann–Whitney tests were used as the primary analysis, with Welch *t* test on log_10_-transformed data performed as sensitivity analysis (see “Methods”). Group comparisons in patients (**H**, **I**, **M**, and **N**) were analyzed using two-tailed Mann–Whitney tests. Correlation analyses in human cohorts (**J**, **K**, **O**, and **P**) were performed using two-tailed Spearman rank correlation. ROC analysis (**L**) was used to compare biomarker performance for discrimination of cachectic vs. noncachectic patients with colorectal cancer. Kaplan–Meier survival curves (**Q** and **R**) were compared using the log-rank (Mantel–Cox) test. Significance is denoted as *, *P* < 0.05; ***, *P* < 0.001; ****, *P* < 0.0001; ns, not significant.

In the colon adenocarcinoma C26 cachexia model (23), plasma ADAMTSL4 levels significantly increased compared with those in mice bearing noncachexigenic MC38 tumors or phosphate-buffered saline (PBS)-injected controls (Fig. 1A and B). Similarly, *Apc*^Min/+^ mice, a genetic model of intestinal tumorigenesis with cachexia (24), exhibited elevated circulating ADAMTSL4 relative to littermate controls (Fig. 1C and D). Consistent results were observed in Lewis lung carcinoma (LLC) tumor-bearing mice, another cachexia model (23), with increased ADAMTSL4 levels compared with non–tumor-bearing controls (Fig. 1E and F). Across all models, plasma ADAMTSL4 showed a significant inverse correlation with body weight change (Fig. 1G), suggesting a functional role for tumor-derived ADAMTSL4 in cancer cachexia.

To determine whether tumor cells are a source of ADAMTSL4, we analyzed conditioned media (CM) from cachexia-inducing cancer cell lines. CM from cachexia-inducing C26 cells induced reduced C2C12 myotube diameter compared with nonconditioned medium (NCM) or CM from non–cachexia-inducing MC38 cells (Supplementary Fig. S1A and S1B). In addition, CM from C26 cells increased glycerol release from differentiated 3T3-L1 adipocytes (Supplementary Fig. S1C). Notably, ADAMTSL4 levels were elevated in CM from C26 cells compared with MC38 cells (Supplementary Fig. S1D). Consistent with these findings, CM from cachexia-inducing LLC cells reduced C2C12 myotube diameter (Supplementary Fig. S1E and S1F), increased glycerol release from differentiated 3T3-L1 adipocytes (Supplementary Fig. S1G), and exhibited elevated ADAMTSL4 levels (Supplementary Fig. S1H). Similarly, pancreatic ductal adenocarcinoma (PDAC) cell lines with high ADAMTSL4 secretion induced myotube and adipocyte wasting (Supplementary Fig. S1I–S1L), linking elevated ADAMTSL4 secretion to cachexia-inducing potential.

### Circulating ADAMTSL4 Is Associated with Cachexia Severity in Patients with Cancer

We next evaluated the clinical relevance of our preclinical findings by examining circulating ADAMTSL4 levels in different cohorts of patients with cancer. Specifically, we analyzed plasma samples from noncachectic (non–cancer cachexia) patients and from patients with cancer cachexia diagnosed with various tumor types. In accordance with Fearon’s consensus definition, cachectic patients were defined as those with weight loss >5% or >2% in individuals with features of wasting, such as a body mass index (BMI) below 20 kg/m^2^ or sarcopenia (25).

In patients with colorectal cancer (Supplementary Table S1), cachectic individuals exhibited significantly greater relative body weight loss (Fig. 1H) and significantly elevated circulating ADAMTSL4 levels compared with non–cancer cachexia patients (Fig. 1I). Consistent with this, continuous analyses revealed a modest but significant inverse correlation between ADAMTSL4 and relative body weight change (Fig. 1J) and a modest but significant positive correlation with cachexia grade (Fig. 1K), indicating a graded association with cachexia severity. In addition, circulating ADAMTSL4 levels were associated with systemic metabolic alterations. Specifically, ADAMTSL4 levels showed a negative correlation with BMI (Supplementary Fig. S1M), were observed in the context of an increased C-reactive protein (CRP)/albumin ratio in cachectic patients (Supplementary Fig. S1N), and negatively correlated with hemoglobin levels (Supplementary Fig. S1O), a marker of cachexia severity (26, 27). These findings support a link between circulating ADAMTSL4 and systemic metabolic impairment.

To position ADAMTSL4 relative to established cachexia-associated biomarkers, we performed receiver operating characteristic (ROC) analysis in patients with colorectal cancer (Fig. 1L). ADAMTSL4 demonstrated modest but statistically significant classification performance (AUROC = 0.6638, *P* = 0.0458), outperforming CRP, albumin, and TGFβ1 and showing comparable performance to the CRP/albumin ratio. As expected, GDF15 showed the strongest discriminatory capacity (AUROC = 0.7941, *P* = 0.0012), consistent with its established role as a canonical cachexia mediator. Consistent with this, circulating GDF15 levels were significantly elevated in cachectic patients and positively correlated with cachexia severity (Supplementary Fig. S1P and S1Q), whereas circulating TGFβ1 levels did not show a statistically robust association with cachexia status and exhibited only weak, nonsignificant relationships with cachexia-related parameters (Supplementary Fig. S1P). These findings are consistent with the known biology of TGFβ, which is primarily regulated through local activation rather than circulating abundance (28).

To further validate these findings, we analyzed an independent cohort of patients with NSCLC from the Tracking Cancer Evolution through Therapy (TRACERx) study (29). Upon stratification by histologic subtype, elevated circulating ADAMTSL4 levels were observed in cachectic patients with lung adenocarcinoma (LUAD) compared with noncachectic individuals (Fig. 1M and N; Supplementary Table S2). In this subtype-restricted cohort, higher ADAMTSL4 levels were significantly associated with greater relative body weight loss (Fig. 1O) and increased cachexia severity (Fig. 1P), consistent with the observations in patients with colorectal cancer. ADAMTSL4 levels were quantified using an Olink proximity extension assay and are reported as normalized protein expression (NPX) values (log_2_ scale); although not directly comparable with enzyme-linked immunosorbent assay (ELISA)-based measurements, the direction and strength of the associations were consistent across cohorts. Given the association between cachexia and poor clinical outcomes (18, 21), we next assessed the relationship between circulating ADAMTSL4 levels and survival. In the broader NSCLC cohort, patients stratified into high- versus low-ADAMTSL4 groups (median NPX cutoff) showed significantly reduced OS in the high-ADAMTSL4 group (Fig. 1Q), whereas no significant association was observed with disease-free survival (Fig. 1R). Because the strongest cachexia-related association was observed in LUAD, the LUAD-restricted OS analysis is additionally provided in Supplementary Fig. S1R. This overall pattern suggests that ADAMTSL4 is more closely associated with systemic wasting phenotypes than tumor recurrence, similar to other cachexia-linked factors such as GDF15 (14, 29).

Together, these findings demonstrate that circulating ADAMTSL4 is associated with cancer cachexia severity across multiple preclinical models and in subtype-refined human cohorts, particularly colorectal cancer and LUAD, supporting its role as a tumor-derived mediator of cancer-associated wasting.

### ADAMTSL4 Overexpression Transforms MC38 Cells to Induce Cachexia

To test whether tumor-derived ADAMTSL4 is sufficient to promote wasting, we transduced non–cancer cachexia-inducing MC38 cells with *GFP*- or *Adamtsl4*-expressing lentiviruses, generating MC38-GFP and MC38-ADAMTSL4 lines (Supplementary Fig. S2A). MC38-ADAMTSL4 cells showed increased ADAMTSL4 levels in whole-cell lysates (Supplementary Fig. S2B and S2C) and CM (Supplementary Fig. S2D), confirmed by ELISA, without affecting *in vitro* cell growth (Supplementary Fig. S2E).

CM from MC38-ADAMTSL4 cells reduced C2C12 myotube diameter relative to CM from MC38-GFP or NCM (Supplementary Fig. S2F and S2G). Cancer cachexia–associated muscle wasting involves activation of proteolytic systems, including the ubiquitin–proteasome and autophagy–lysosomal pathways (30, 31). Accordingly, MC38-ADAMTSL4 CM increased expression of *Trim63*, an E3-ubiquitin ligase involved in proteasomal proteolysis, as well as *Atg5* and *Map1lc3*, markers of autophagy, in myotubes (Supplementary Fig. S2H), consistent with activation of catabolic signaling. Moreover, ADAMTSL4-enriched CM promoted lipolysis in differentiated 3T3-L1 adipocytes (Supplementary Fig. S2I) and increased adipocyte triglyceride lipase (ATGL; Supplementary Fig. S2J), a key enzyme in triglyceride hydrolysis (32).

To further validate these findings, we generated HEK293T-ADAMTSL4 and control HEK293T-GFP cells as a heterologous expression system. ADAMTSL4 overexpression increased protein secretion without affecting cell growth (Supplementary Fig. S2K and S2L), and CM from these cells induced C2C12 myotube atrophy, increased atrophy gene expression, and promoted lipolysis in 3T3-L1 adipocytes (Supplementary Fig. S2M and S2O).

**Figure 2.**
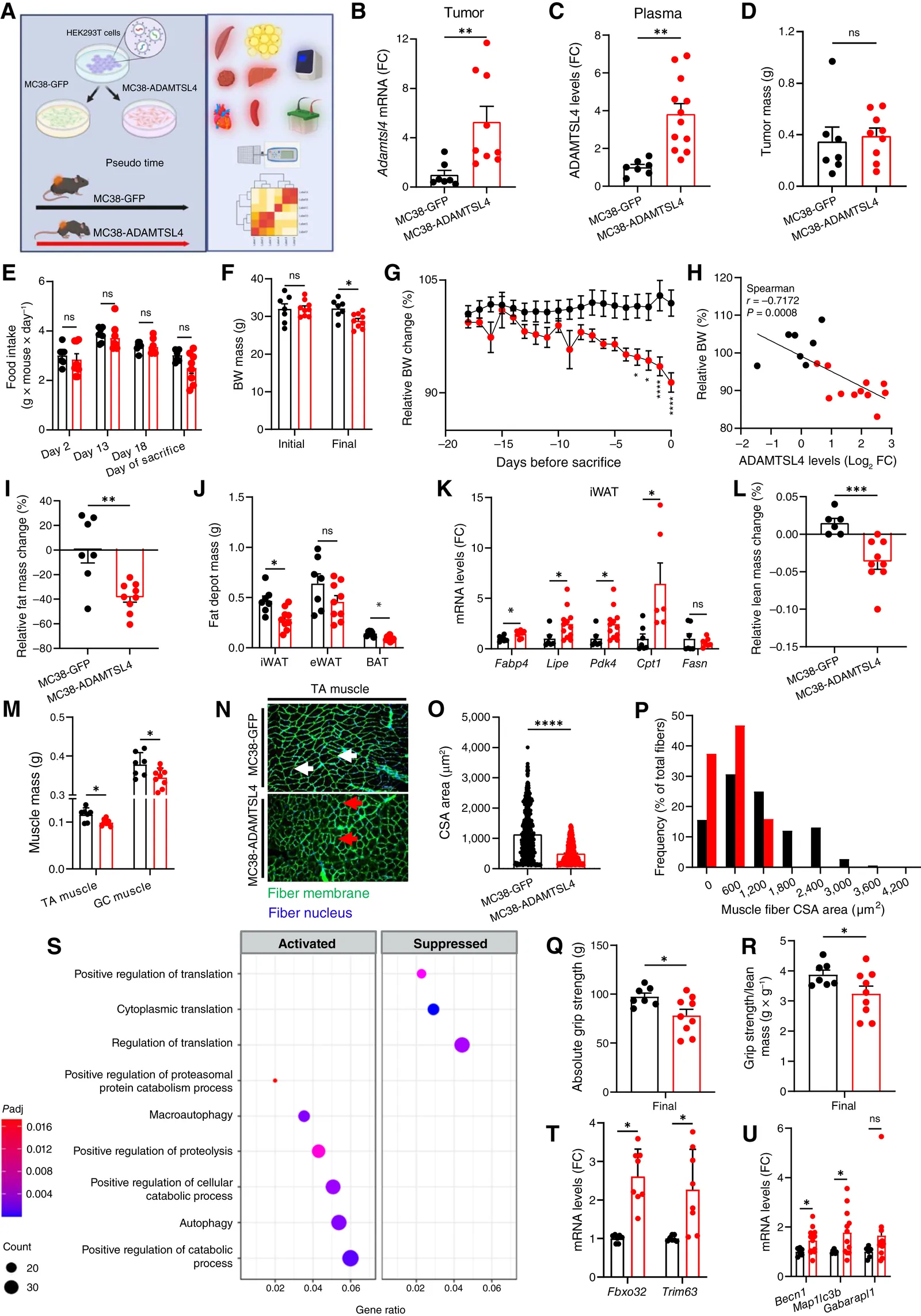
Tumor-specific overexpression of ADAMTSL4 is sufficient to drive cancer cachexia. **A–U,** Mice were subcutaneously inoculated with control MC38-GFP cells (*n* = 7) or MC38 cells stably overexpressing ADAMTSL4 (MC38-ADAMTSL4; *n* = 9–12). **A,** Schematic of the preclinical model used to assess the cachexia-inducing capacity of MC38 tumor-derived ADAMTSL4. **B,***Adamtsl4* mRNA levels in tumors. **C,** Plasma ADAMTSL4 protein levels determined by Western blot analysis. **D,** Tumor mass. **E,** Food intake in tumor-bearing mice. Food intake was measured daily by weighing chow pellets at a fixed time point. Mice were housed two per cage, and total cage consumption was calculated as the difference between food provided and food remaining on the following day, with visual inspection to account for potential spillage. Intake was normalized to the number of mice per cage and expressed as grams per mouse per day. No significant differences were observed between groups across the study period. **F,** Initial and final carcass weight (tumor-free body weight). **G,** Longitudinal kinetics of relative body weight (BW) change (%) in the days preceding sacrifice. **H,** Spearman correlation between plasma ADAMTSL4 levels and final relative BW change (*n* = 18 XY pairs). Relative BW change was calculated from the longitudinal measurements shown in **G**. **I,** Relative change in fat mass measured by EchoMRI. **J,** Fat mass in distinct adipose depots: iWAT, eWAT, and BAT. **K,** Expression of lipid metabolism-related genes in iWAT assessed by qPCR. **L,** Relative change in lean mass measured by EchoMRI. **M,** TA and GC muscle weights. **N,** Representative images of TA muscle cross-sections stained with WGA Alexa Fluor 488 and DAPI (200× magnification; scale bar, 100 μm). Representative structural features are highlighted by arrows (white, control; red, ADAMTSL4-overexpressing condition). **O,** Quantification of muscle fiber CSA. **P,** Frequency distribution of fiber CSA in TA muscle. **Q,** Absolute grip strength (forelimbs and hindlimbs). **N–P,** Complementary readouts derived from the same TA muscle cross-section analysis. **R,** Grip strength normalized to lean mass (g/g). **S,** Selected GO terms identified by GSEA in GC muscle from MC38-ADAMTSL4 vs. MC38-GFP tumor-bearing mice (see also Supplementary Data S1 and S2 for full GO term analysis results). **T,** Validation of marker genes associated with proteasomal catabolic processes (*Fbxo32* and *Trim63*). **U,** Validation of macroautophagy-related genes (*Becn1*, *Map1lc3*, and *Gabarapl1*) by qPCR. Data are presented as the mean ± SEM. Each dot represents an individual biological replicate. *Statistical analysis*: two-tailed unpaired *t* test (**B–D**, **F**, **I–M**, **O**, **Q**, **R**, **T**, and **U**), Spearman rank correlation (**H**), two-way mixed-effects analysis with Šídák multiple-comparisons test (**E** and **G**), and weighted Kolmogorov–Smirnov test for GSEA enrichment scores (**S**). Significance is denoted as *, *P* < 0.05; **, *P* < 0.01; ***, *P* < 0.001; ****, *P* < 0.0001; ns, not significant. [**A,** Created in BioRender. Machado, J. (2026) https://BioRender.com/q1z1l3n.]

To verify our findings *in vivo*, we subcutaneously implanted MC38-ADAMTSL4 or control MC38-GFP cells into 6-month-old male C57BL/6N mice (Fig. 2A). *Adamtsl4*-overexpressing tumors exhibited robust gene expression and elevated circulating ADAMTSL4 protein (Fig. 2B and C; Supplementary Fig. S2P) without differences in tumor burden (Fig. 2D). Food intake did not differ between groups across the study period (Fig. 2E), despite progressive loss of body weight, lean mass, and adipose tissue in ADAMTSL4-expressing tumor-bearing mice. Thus, anorexia was not evident in this model, although dedicated metabolic cage studies will be required to exclude more subtle differences in energy intake or expenditure. Notably, both groups exhibited comparable initial carcass weight, whereas final carcass weight was significantly reduced in MC38-ADAMTSL4–bearing mice (Fig. 2F), indicating a tumor-driven reduction in body mass independent of baseline differences. Longitudinal analysis further revealed a progressive decrease in relative body weight in MC38-ADAMTSL4–bearing mice compared with MC38-GFP controls (Fig. 2G). Importantly, circulating ADAMTSL4 levels showed a significant inverse correlation with relative body weight change (Fig. 2H), supporting a quantitative relationship between tumor-derived ADAMTSL4 and cachexia severity. Body composition analysis revealed significant total fat loss (Fig. 2I) and depot-specific adipose tissue reduction (Fig. 2J). This was accompanied by upregulation of *Lipe*, encoding hormone-sensitive lipase, and fatty acid oxidation genes (*Pdk4* and *Cpt1b*), without induction of *Fasn* (Fig. 2K), supporting enhanced lipolytic and oxidative metabolism, consistent with the prowasting effects observed *in vitro*.

Skeletal muscle atrophy is a hallmark of cancer cachexia (1, 19). MC38-ADAMTSL4 tumors induced a marked reduction in lean mass (Fig. 2L), whereas liver, spleen, and heart weights remained unchanged (Supplementary Fig. S2Q), indicating a selective wasting phenotype. *Adamtsl4* mRNA was not induced in the peripheral tissues analyzed and showed tissue-specific variation, including reduced expression in gastrocnemius (GC) muscle (Supplementary Fig. S2R), supporting the interpretation that the tumor is the primary source of circulating ADAMTSL4 in this model. Consistent with this, tibialis anterior (TA) and GC muscle weights were significantly reduced in ADAMTSL4-expressing tumor-bearing mice (Fig. 2M), corresponding to ∼14.5% and ∼8.9% decreases, respectively. Wheat germ agglutinin (WGA) staining of TA sections revealed reduced myofiber cross-sectional area (CSA) and a shift toward smaller fibers (Fig. 2N–P), classic features of muscle atrophy. These structural changes translated into functional impairment, as indicated by reduced grip strength (Fig. 2Q and R).

To dissect the molecular programs driving ADAMTSL4-induced muscle atrophy, we performed RNA sequencing (RNA-seq) on GC muscles from cachectic and control animals. Gene ontology (GO) analysis of differentially expressed genes (DEG) showed enrichment for proteolytic and catabolic pathways, including “positive regulation of proteolysis,” “proteasomal protein catabolic process,” and “autophagy” (Fig. 2S; Supplementary Data S1 and S2). These signatures were corroborated by increased expression of *Fbxo32* and *Trim63* (atrogenes) and autophagy-related genes *Becn1* and *Map1lc3* (Fig. 2T and U), indicating concurrent activation of ubiquitin–proteasome and autophagy–lysosome systems.

Together, these results demonstrate that tumor-derived ADAMTSL4 is sufficient to drive cancer cachexia *in vivo*, promoting coordinated wasting of adipose tissue and skeletal muscle through conserved catabolic programs. These findings support a functional role for ADAMTSL4 as a systemic mediator of tissue loss in cancer cachexia.

### Tumor-Derived ADAMTSL4 Is Required for Cancer Cachexia and Muscle Wasting

To assess whether ADAMTSL4 is required for the cachexia-inducing activity of C26 cells, we generated stable knockdown lines using lentiviral short hairpin RNAs (shRNA), yielding C26-shCtrl and C26-shAdamtsl4 cells (Supplementary Fig. S3A). Clone shAdamtsl4 #3 achieved efficient suppression of *Adamtsl4* mRNA, protein, and secreted ADAMTSL4 (Supplementary Fig. S3B–S3E) without affecting cell proliferation (Supplementary Fig. S3F). CM from C26-shAdamtsl4 cells elicited significantly reduced C2C12 myotube atrophy compared with CM from C26-shCtrl (Supplementary Fig. S3G and S3H), accompanied by suppression of *Trim63*, *Fbxo32*, and autophagy–lysosomal gene expression (Supplementary Fig. S3I). Transient siRNA-mediated *Adamtsl4* knockdown recapitulated these effects without altering cell proliferation (Supplementary Fig. S3J–S3L), confirming a direct role for tumor-secreted ADAMTSL4 in myotube wasting. Extending these findings to the pancreatic adenocarcinoma line 8025, which secretes high ADAMTSL4 levels (Supplementary Fig. S1I–S1L), stable *Adamtsl4* knockdown (8025-shAdamtsl4) reduced mRNA expression without affecting proliferation (Supplementary Fig. S3M and S3N) and similarly attenuated CM-induced myotube atrophy and catabolic gene expression (Supplementary Fig. S3O and S3P). Together, these *in vitro* data support a functional role for ADAMTSL4 in tumor-driven muscle wasting.

**Figure 3.**
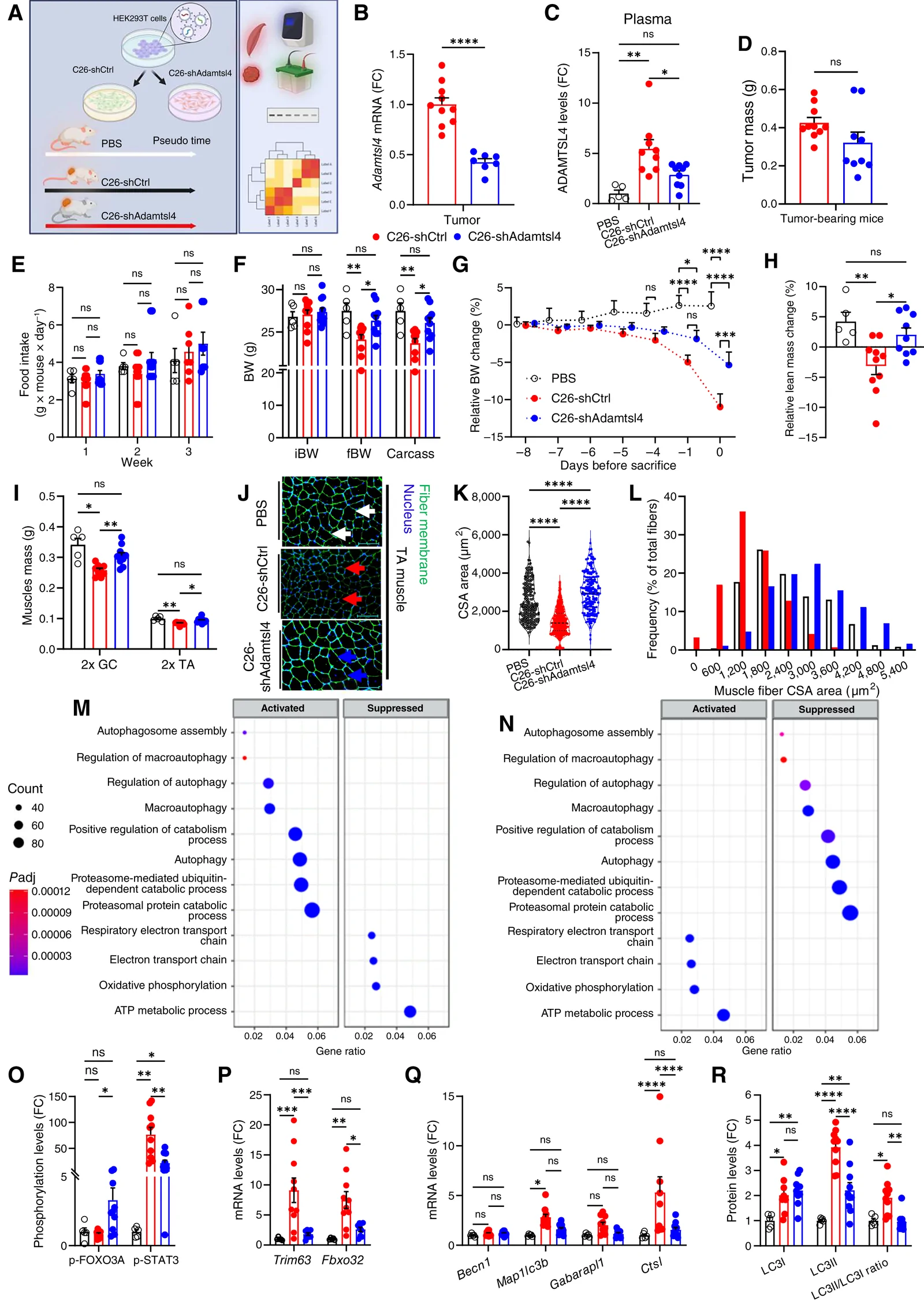
Suppression of C26 tumor-derived ADAMTSL4 attenuates cancer cachexia and muscle atrophy *in vivo*. **A–R,** Mice were subcutaneously inoculated with PBS (*n* = 5), control C26 cells (C26-shCtrl, *n* = 10), or C26 cells with stable ADAMTSL4 knockdown (C26-shAdamtsl4, *n* = 9). **A,** Schematic overview of the preclinical model used to investigate the role of C26 tumor-derived ADAMTSL4 in cachexia. **B,** Adamtsl4 mRNA expression in tumors assessed by qPCR. **C,** Plasma ADAMTSL4 protein levels measured by Western blot. **D,** Tumor mass and (**E**) food intake in tumor-bearing or PBS-injected mice. Food intake measured daily per cage and normalized to grams per mouse per day. Mice were housed two per cage unless otherwise indicated. **F,** Initial body weight (iBW), final BW (fBW), and tumor-free carcass weight. **G,** Relative BW loss kinetics during the final days prior to sacrifice. **H,** Relative change in lean mass determined by EchoMRI. **I,** GC and TA muscle weights. **J,** Representative images of TA muscle sections stained with WGA Alexa Fluor 488 and DAPI (200× magnification; scale bar, 100 μm). Representative structural features are highlighted by arrows (white, PBS/control; red, cachectic condition; blue, ADAMTSL4 knockdown). **K,** Quantification of muscle fiber CSA (*n* = 3 per group, >500 fibers per mouse) and (**L**) fiber size distribution in TA. **M,** Selected GO terms from GSEA of GC muscle from C26-shCtrl vs. PBS-injected mice (see also Supplementary Data S3 and S4 for full GO term analysis results). **N,** Selected GO terms from GSEA of GC muscle from C26-shAdamtsl4 vs. C26-shCtrl tumor-bearing mice (see also Supplementary Data S5 and S6 for full GO term analysis results). **O,** Quantification of Western blot analysis of key proteins involved in proteasomal catabolism and autophagy, including p-FOXO3a at T32 and p-STAT3 at Y705. **P,** mRNA expression of proteasomal catabolic genes (*Fbxo32* and *Trim63*) and (**Q**) autophagy-related genes (*Becn1*, *Map1lc3*, and *Gabarapl1*) validated by qPCR. **R,** Quantification of Western blot analysis of autophagy marker LC3, showing LC3-I, LC3-II, and the LC3-II/LC3-I ratio. Data are presented as the mean ± SEM. Each dot represents an individual biological replicate. Statistical analysis: two-tailed unpaired *t* test with Welch correction (**B** and **D**), one-way ANOVA with Tukey multiple-comparisons test (**C**, **F**, **G–I**, **K**, and **O–R**), two-way ANOVA with mixed-effects model (REML) followed by Tukey’s test (**E** and **G**), and GSEA enrichment scores calculated using weighted Kolmogorov–Smirnov statistics (**M** and **N**). Significance is denoted as *, *P* < 0.05; **, *P* < 0.01; ***, *P* < 0.001; ****, *P* < 0.0001; ns, not significant. [**A,** Created in BioRender. Machado, J. (2026) https://BioRender.com/vx8zzgy.]

To test the role of tumor-derived ADAMTSL4 *in vivo*, we implanted C26-shCtrl or C26-shAdamtsl4 cells subcutaneously into 12-week-old male mice, alongside PBS-injected controls (Fig. 3A). C26-shAdamtsl4 tumors showed markedly reduced *Adamtsl4* mRNA (Fig. 3B) and lower circulating ADAMTSL4 protein levels compared with controls (Fig. 3C; Supplementary Fig. S3Q), without affecting tumor mass (Fig. 3D), apoptosis marker expression (*Pmaip1*; Supplementary Fig. S3R), or liver and spleen weights (Supplementary Fig. S3S). Consistent with the gain-of-function model, food intake did not differ between groups across the study period (Fig. 3E), indicating that the protective effect of *Adamtsl4* suppression on body weight loss was not explained by altered caloric intake. Mice bearing C26-shAdamtsl4 tumors exhibited markedly attenuated body weight loss (Fig. 3F and G) and were protected from lean mass depletion and skeletal muscle atrophy (Fig. 3H and I). Histology revealed full preservation of TA myofiber CSA (Fig. 3J and K) and normalized fiber size distribution (Fig. 3L), in contrast to pronounced wasting in C26-shCtrl tumor-bearing mice.

Together, these results demonstrate that tumor-derived ADAMTSL4 is required for the development of cancer cachexia and associated skeletal muscle atrophy in the C26 preclinical model.

### Tumor-Derived ADAMTSL4 Drives Activation of Muscle Catabolic Programs

To investigate the molecular pathways underlying ADAMTSL4-driven muscle atrophy, we performed transcriptomic profiling of GC muscles from PBS-injected controls and cachectic C26-shCtrl tumor-bearing mice. GO enrichment of DEGs revealed upregulation of key catabolic programs, including “proteasomal protein catabolic process,” “positive regulation of catabolic process,” and “autophagy,” consistent with cachexia-associated muscle wasting (Fig. 3M; Supplementary Data S3 and S4). To determine the contribution of tumor-derived ADAMTSL4 to these transcriptional changes, we compared GC transcriptomes between C26-shCtrl and C26-shAdamtsl4 tumor-bearing mice. Suppression of tumor-derived ADAMTSL4 reversed the transcriptional activation of atrophy-associated pathways. GO analysis showed coordinated downregulation of “positive regulation of catabolic process,” “proteasome-mediated ubiquitin-dependent protein catabolic process,” and “macroautophagy” in muscles of C26-shAdamtsl4-bearing animals (Fig. 3N; Supplementary Data S5 and S6), consistent with preserved muscle mass.

These transcriptomic changes were corroborated at the protein level. Western blotting revealed increased phosphorylation of FoxO3a at threonine 32, an inhibitory modification that attenuates transcriptional activity, alongside decreased phosphorylation of STAT3 at tyrosine 705 (Fig. 3O; Supplementary Fig. S3T), both central regulators of atrophy- and autophagy-related programs during cachexia. Concordantly, expression of FOXO3A and STAT3 target genes, including *Trim63*, *Fbxo32*, *Map1lc3*, and *Ctsl*, was reduced in GC muscle from C26-shAdamtsl4 mice (Fig. 3P and Q), together with suppression of autophagy markers (Fig. 3R; Supplementary Fig. S3T), reinforcing the role of ADAMTSL4 in catabolic remodeling.

Together, these results establish tumor-derived ADAMTSL4 as a regulator of muscle catabolic remodeling, coordinating transcriptional and signaling programs converging on proteasomal degradation and autophagy.

### Tumor-Derived ADAMTSL4 Drives Fat Wasting in Cancer Cachexia

**Figure 4.**
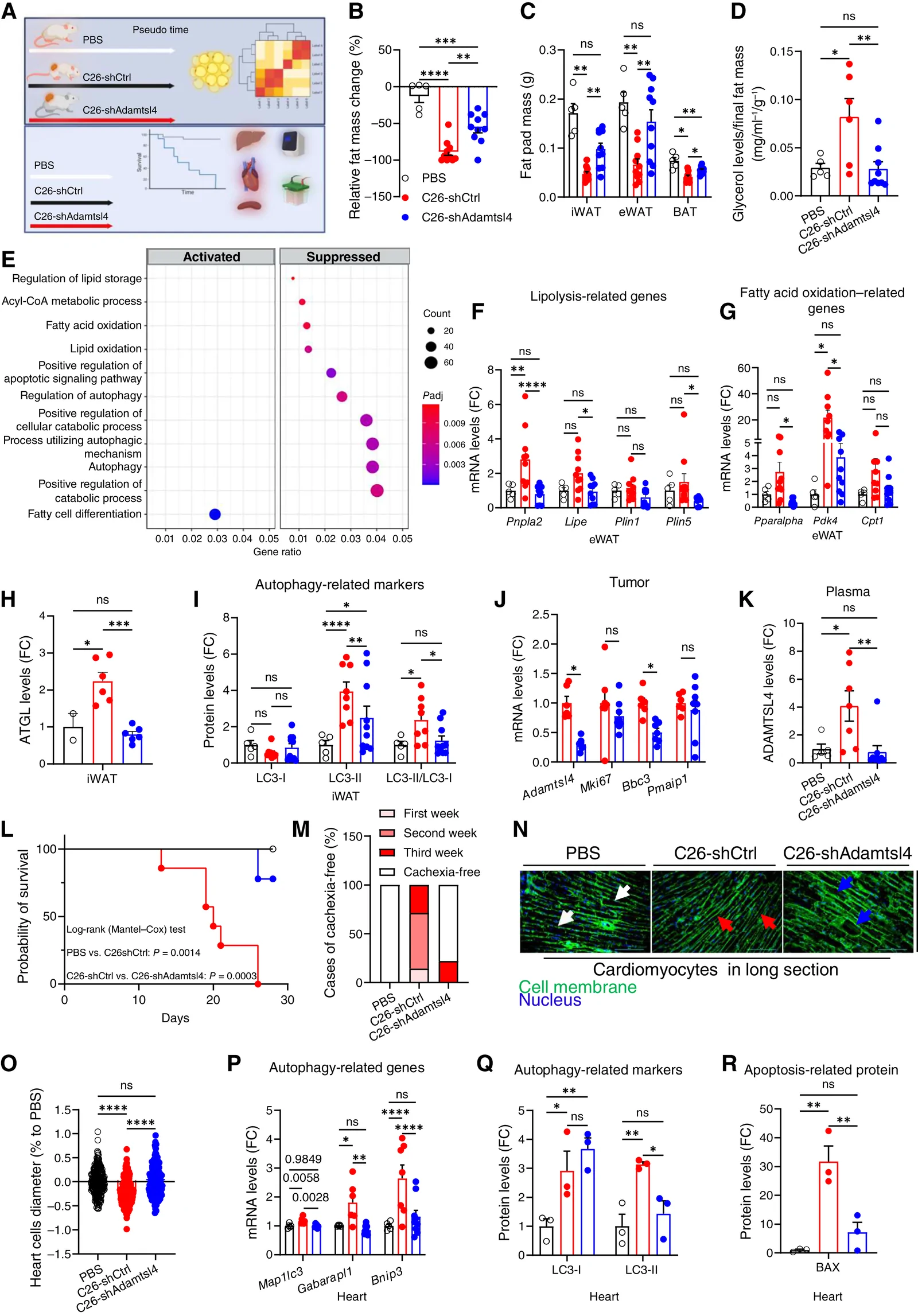
Stable knockdown of tumor-derived ADAMTSL4 attenuates adipose tissue wasting, prolongs cachexia-related survival, and protects against cancer-induced cardiac atrophy. **A–R,** Mice were subcutaneously injected with PBS, control C26 cells (C26-shCtrl), or ADAMTSL4-depleted C26 cells (C26-shAdamtsl4). **A,** Schematic overview of the preclinical model used to assess the impact of tumor-derived ADAMTSL4 on adipose wasting, survival, and cardiac atrophy. **B,** Relative fat mass changes measured by EchoMRI. **C,** Mass of individual fat depots: iWAT, eWAT, and BAT. **D,** Plasma glycerol levels normalized to final fat mass. **E,** Selected GO terms from GSEA of eWAT comparing C26-shAdamtsl4 vs. C26-shCtrl tumor-bearing mice (see also Supplementary Data S7 and S8 for full GO term analysis results). **F,** mRNA expression of lipolysis-related genes and (**G**) fatty acid oxidation genes in eWAT, validating GSEA-enriched “fatty acid oxidation” and “lipid oxidation” terms. **H,** Western blot analysis of ATGL protein levels. **I,** Western blot analysis of autophagy-related proteins in eWAT, supporting the “autophagy” GO term from GSEA. **J–R,** Mice were subcutaneously inoculated with PBS (*n* = 5), C26-shCtrl cells (*n* = 6–7), or C26-shAdamtsl4 cells (*n* = 6–9). **J,** Tumor mRNA expression of *Adamtsl4*, *Mki67*, *Bbc3*, and *Pmaip1* assessed by qPCR. **K,** Plasma ADAMTSL4 protein levels measured by Western blot. **L,** Kaplan–Meier curve showing percentage of mice developing cachexia over time. **M,** Percentage of cachexia-free mice at endpoint. **N,** Representative heart sections stained with WGA Alexa Fluor 488 and DAPI (200× magnification; scale bar, 100 μm). Representative structural features are highlighted by arrows (white, PBS/control; red, cachectic condition; blue, ADAMTSL4 knockdown). **O,** CSA quantification of cardiomyocytes (*n* = 3 per group, >200 cells per mouse). **P,** Cardiac expression of autophagy-related genes (*Bnip3*, *Map1lc3*, and *Gabarapl1*) by qPCR. **Q,** Western blot analysis of cardiac autophagy (LC3-I and LC3-II) and (**R**) apoptosis (BAX) markers. Data are presented as the mean ± SEM. Each dot represents an individual biological replicate. Statistical analysis: one-way ANOVA with Tukey multiple-comparisons test (**B–D**, **F–I**, **K**, and **O**), multiple two-tailed unpaired *t* tests with Welch correction (**J**), two-way ANOVA or mixed-effects model (REML) with Tukey multiple-comparisons test (**P–R**), and GSEA enrichment scores calculated using weighted Kolmogorov–Smirnov statistics (**E**). Significance is denoted as *, *P* < 0.05; **, *P* < 0.01; ***, *P* < 0.001; ****, *P* < 0.0001; ns, not significant. [**A,** Created in BioRender. Machado, J. (2026) https://BioRender.com/79ygt3l.]

We next examined the established C26 *Adamtsl4*-knockdown model in the context of adipose tissue wasting (Fig. 4A). To assess the role of tumor-derived ADAMTSL4 in adipose tissue wasting, we first tested its effects *in vitro*. Treatment of mature 3T3-L1 adipocytes with CM from C26-shCtrl cells induced lipolysis and upregulation of ATGL (Supplementary Fig. S4A–S4C). In contrast, CM from C26-shAdamtsl4 reduced both lipolysis and ATGL levels, supporting a role for ADAMTSL4 in pro-lipolytic activity. Acute siRNA-mediated *Adamtsl4* knockdown in C26 cells similarly blunted the lipolytic effect of CM (Supplementary Fig. S4D–S4F). These findings were confirmed in the pancreatic adenocarcinoma 8025 line, in which stable ADAMTSL4 depletion reduced CM-induced adipocyte lipolysis (Supplementary Fig. S4G).

To test whether ADAMTSL4 is sufficient to trigger adipocyte wasting, we generated recombinant human ADAMTSL4 C-terminal protein (rhADAMTSL4-CT) containing the last five thrombospondin type 1 repeats and the PLAC domain, fused to BFP, V5, and His tags (Supplementary Fig. S4H and S4I). Based on structural homology to ADAMTSL6 (33, 34), ADAMTSL4-CT is predicted to retain functional activity. Treatment of 3T3-L1 adipocytes with ADAMTSL4-CT induced dose-dependent increases in lipolysis, with 250 ng/mL eliciting a maximal effect, comparable with levels in tumor CM and plasma of cachectic mice (Fig. 1B, D, and F; Supplementary Figs. S1D and S4J). Similar effects were observed in hTERT-immortalized human MSC-derived adipocytes (Supplementary Fig. S4K), supporting the relevance of ADAMTSL4 in adipocyte catabolism.

We next evaluated the *in vivo* impact of tumor-derived ADAMTSL4 on fat wasting. Subcutaneous implantation of C26-shCtrl cells into 12-week-old male mice caused substantial reduction in total fat mass and depot-specific inguinal (iWAT), epididymal (eWAT), and brown adipose tissue (BAT), accompanied by elevated lipolysis (Fig. 4B–D). By contrast, these cachexia-associated changes were largely prevented in mice bearing C26-shAdamtsl4 tumors, with normalization of circulating glycerol levels relative to fat mass (Fig. 4B–D).

RNA-seq analysis of eWAT revealed that Adamtsl4 knockdown suppressed key catabolic programs, including “autophagy,” “positive regulation of cellular catabolic processes,” and “positive regulation of apoptotic signaling” (Fig. 4E; Supplementary Data S7 and S8). Fatty acid and lipid oxidation pathways were also downregulated. Consistent with this, induction of lipolytic enzymes *Pnpla2* and *Lipe* (Fig. 4F) and fatty acid oxidation genes *Ppara* and *Pdk4* (Fig. 4G) was blunted in eWAT from C26-shAdamtsl4 mice. At the protein level, ATGL levels were reduced in iWAT (Fig. 4H; Supplementary Fig. S4L). Autophagic activity, assessed via LC3 processing, was increased in cachectic mice but normalized upon *Adamtsl4* knockdown (Fig. 4I; Supplementary Fig. S4M).

Together, these results indicate that tumor-derived ADAMTSL4 is required for adipose tissue wasting in cancer cachexia by promoting lipolysis, fatty acid oxidation, and autophagy, highlighting its role in systemic tissue catabolism.

### Suppression of *Adamtsl4* in C26 Tumors Prolonged Cachexia-Free Survival and Attenuated Cardiac Atrophy

Cachexia is a major determinant of mortality in patients with gastrointestinal cancer (35) and in the C26 mouse model (36). To assess whether tumor-derived ADAMTSL4 affects cachexia-free survival, C26 tumor-bearing mice were monitored until ∼15% body weight loss, indicative of refractory cachexia and serving as the humane endpoint (Fig. 4A). Suppression of *Adamtsl4* in C26 tumors did not affect tumor mass (Supplementary Fig. S4N) or proliferation- and apoptosis-related gene expression (Fig. 4J). C26-shAdamtsl4 tumor-bearing mice exhibited markedly reduced tumor *Adamtsl4* mRNA (Fig. 4J) and circulating ADAMTSL4 protein levels (Fig. 4K; Supplementary Fig. S4O). Cachexia, defined as >5% body weight loss, was observed around day 8 in C26-shCtrl mice, with all animals reaching the humane endpoint within 2 to 3 weeks due to severe weight loss (>12 to 17%; Fig. 4L and M; Supplementary Fig. S4P). By contrast, nearly 80% of C26-shAdamtsl4 mice remained cachexia-free throughout the study, with the experiment terminated for reasons related to tumor burden rather than wasting (Fig. 4L and M; Supplementary Fig. S4P), demonstrating that *Adamtsl4* suppression significantly improves cachexia-free survival.

Cardiac wasting is a prominent feature of cachexia and may contribute to cancer mortality (37, 38). Consistent with this, C26-shCtrl tumor-bearing mice exhibited reduced heart mass (Supplementary Fig. S4Q) together with smaller cardiomyocyte diameters (Fig. 4N and O) compared with nontumor controls. In contrast, *Adamtsl4*-deficient tumor-bearing mice were protected from both reductions in heart mass and cardiomyocyte size (Fig. 4N and O; Supplementary Fig. S4Q), indicating preservation of cardiac structure. Whereas expression of muscle atrophy-related ubiquitin ligases remained unchanged (Supplementary Fig. S4R), cardiac wasting was associated with upregulation of autophagy-related genes (Fig. 4P) and increased protein levels of autophagy- and apoptosis-related markers, including LC3-II and BAX, as assessed by Western blot analysis (Fig. 4Q and R; Supplementary Fig. S4S). These molecular alterations were not observed in mice bearing C26-shAdamtsl4 tumors. Although ADAMTSL4 expression was detectable in peripheral tissues affected by cachexia, tumor-specific *Adamtsl4* suppression did not alter its tissue expression pattern (Supplementary Fig. S4T), supporting the conclusion that circulating tumor-derived ADAMTSL4 is the primary source driving the systemic phenotype.

Collectively, these findings suggest that tumor-derived ADAMTSL4 is a key mediator of cachexia-related mortality and systemic wasting, driving coordinated wasting of cardiac, adipose, and skeletal muscle tissues.

### ADAMTSL4 Functionally Engages the Latency-Associated Peptide Domain of Latent TGFβ1

**Figure 5.**
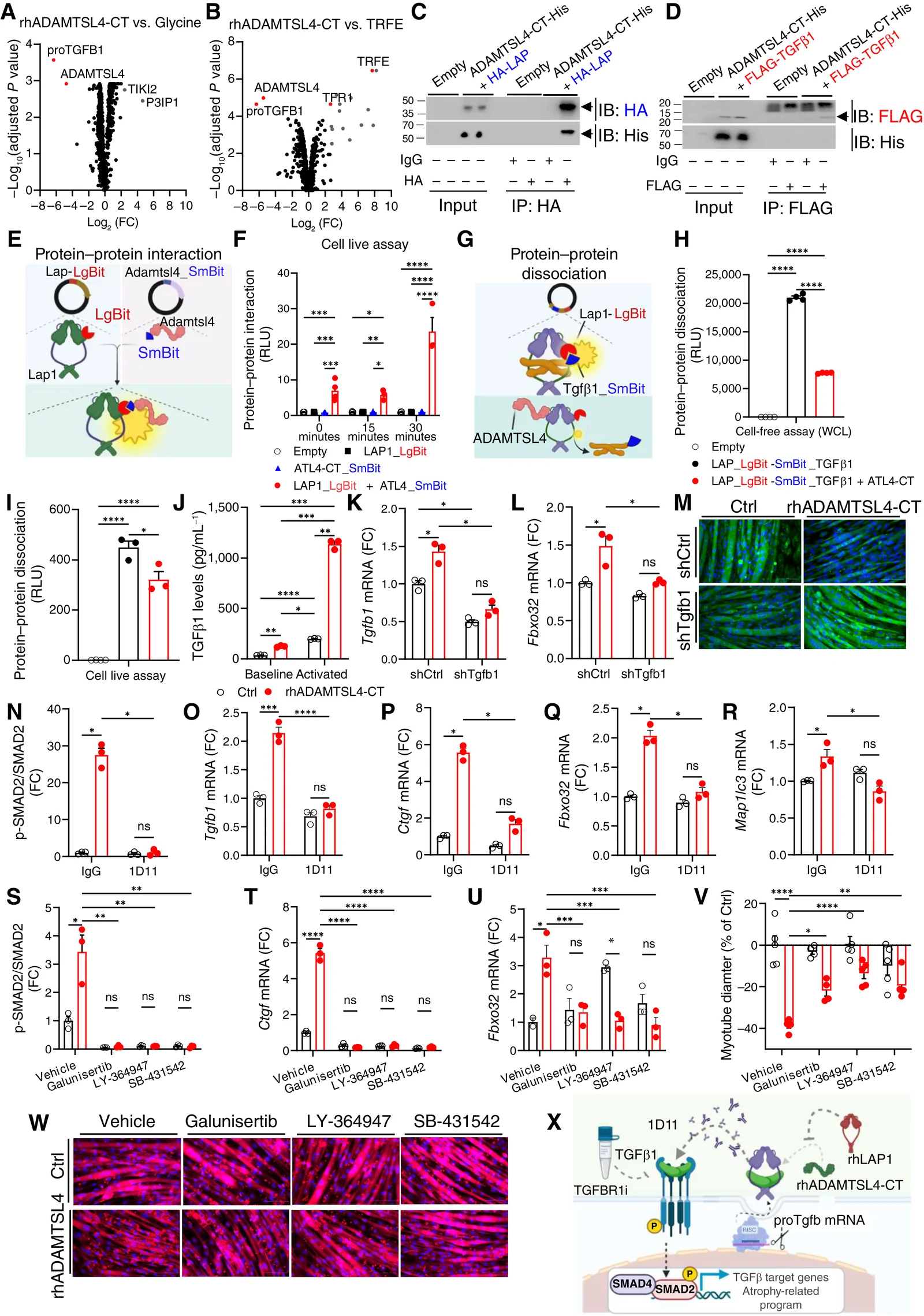
ADAMTSL4 targets LAP of proTGFβ1 to promote myotube atrophy via latency activation and TGFBR1-dependent signaling. **A** and **B,** LRC-TriCEPS experiments were performed using C2C12 myotubes to identify surface interactors of recombinant human ADAMTSL4-CT (rhADAMTSL4-CT). Volcano plots of LC-MS/MS data comparing rhADAMTSL4-CT against glycine (negative control; **A**) or TRFE (positive control; **B**) show enrichment of membrane-associated proteins, including proTGFβ1 (UniProt P04202), as significant targets of rhADAMTSL4-CT (see also Supplementary Table S3 and Supplementary Data S9). **C,** Co-IP assays were performed in HEK293T cells transiently transfected with tagged constructs, followed by IP from whole-cell lysates (WCL). Using HA-tagged LAP (HA-LAP), ADAMTSL4-CT (His- or V5-tagged) was specifically detected in the precipitated complex, demonstrating a direct interaction between ADAMTSL4 and the LAP of proTGFβ1. **D,** Co-IP using FLAG-tagged mature TGFβ1 (FLAG–TGFβ1) did not show interaction with ADAMTSL4-CT, despite robust detection in input controls, indicating that ADAMTSL4 selectively binds LAP but not mature TGFβ1. **E,** Schematic representation of constructs used in NanoBiT luciferase complementation assays. ADAMTSL4 was fused to the small fragment (SmBiT), whereas LAP was fused to the large fragment (LgBiT). Upon protein–protein interaction, proximity of SmBiT and LgBiT enables luciferase reconstitution in the presence of substrate, resulting in luminescence proportional to binding. **F,** NanoBiT assay demonstrating interaction between ADAMTSL4-CT (ATL4-CT) and LAP in live cells. **G,** Schematic representation of the NanoBiT-based dissociation assay. A full-length proTGFβ1 construct was engineered to fuse LgBiT to LAP and SmBiT to the TGFβ1 domain. This design preserves physiologic endoplasmic reticulum (ER)–Golgi processing and proteolytic cleavage while maintaining the noncovalent association of LAP and mature TGFβ1 within the SLC. Disruption of the LAP–TGFβ1 interaction results in separation of LgBiT and SmBiT, leading to decreased luminescence and enabling quantitative assessment of protein–protein dissociation. **H** and **I,** ADAMTSL4 promotes dissociation of mature TGFβ1 from LAP, as measured by reduced NanoBiT luminescence signal. **J,** Total free TGFβ1 protein levels measured by ELISA in supernatants of C2C12 myotubes treated with vehicle or rhADAMTSL4-CT (250 ng/mL). **K** and **L,***Tgfb1* and *Fbxo32* mRNA expression in C2C12 myotubes treated with vehicle or rhADAMTSL4-CT, with or without lentiviral knockdown of proTgfb1. **M,** Representative immunofluorescence images of C2C12 myotubes treated with vehicle or rhADAMTSL4-CT, with or without lentiviral knockdown of proTgfb1. **N,** Western blot analysis of phosphorylated SMAD2 (p-SMAD2) as a readout of TGFβ pathway activation in C2C12 myotubes treated with rhADAMTSL4-CT in the presence or absence of the TGFβ1–neutralizing antibody 1D11 (1.5 μg/mL). **O–R,** qPCR analysis of *Tgfb1*, *Ctgf*, *Fbxo32*, and *Map1lc3* in myotubes treated with rhADAMTSL4-CT ± TGFβ1–neutralizing antibody 1D11 (1.5 μg/mL). **S–X,** Functional inhibition of TGFBR1 using galunisertib (10 μmol/L), LY-364947 (10 μmol/L), or SB-431542 (10 μmol/L) in the context of rhADAMTSL4-CT treatment: (**S**) Western blot analysis of p-SMAD2; (**T** and **U**) *Ctgf* and *Fbxo32* mRNA expression; (**V**) quantification of myotube diameter; (**W**) representative immunofluorescence images. **X,** Schematic summary of the mechanistic framework uncovered in this study. ADAMTSL4 binds the LAP of proTGFβ1, as demonstrated by co-IP and NanoBiT interaction assays. This interaction promotes dissociation of the latent TGFβ1 complex, leading to the release of bioactive TGFβ1. Functionally, ADAMTSL4-induced TGFβ1 activation is inhibited by rhLAP, demonstrating the requirement of LAP engagement. Downstream signaling is blocked by the TGFβ1–neutralizing antibody 1D11, confirming ligand-dependent activation. Pharmacologic inhibition of TGFBR1 (galunisertib, LY-364947, SB-431542) and genetic suppression of proTgfb1 further establish that ADAMTSL4-driven effects on SMAD2 activation and myotube atrophy are mediated through canonical TGFβ signaling. Together, these data support a model in which ADAMTSL4 activates latent TGFβ1 via LAP binding, triggering TGFBR1-dependent signaling and atrophy-related gene programs. Data are presented as the mean ± SEM. Each dot represents an individual biological replicate. *Statistical analysis*: one-way ANOVA with Tukey multiple-comparisons test (**F**, **H**, and **I**), two-way mixed-effects model with Tukey multiple-comparisons test (**J–L** and **N–R**), and two-way mixed-effects model with Holm–Šídák multiple-comparisons test (**S–V**). Significance is denoted as *, *P* < 0.05; **, *P* < 0.01; ***, *P* < 0.001; ****, *P* < 0.0001; ns, not significant. [**E,** Created in BioRender. Machado, J. (2026) https://BioRender.com/xis2eju; **G,** Created in BioRender. Machado, J. (2026) https://BioRender.com/wqwc3bb; **X,** Created in BioRender. Machado, J. (2026) https://BioRender.com/l2gs5ov.]

To elucidate the molecular mechanism underlying ADAMTSL4-driven muscle catabolism, we used ligand-based receptor capture (LRC)-TriCEPS, which enables unbiased identification of surface interactors on live cells under near-physiologic conditions (39). Recombinant human ADAMTSL4-CT, transferrin (TRFE; positive control), or glycine (negative control) were conjugated to the TriCEPS cross-linker and incubated with fully differentiated C2C12 myotubes at 4°C, allowing purification and LC-MS/MS identification of proximal proteins. Of 852 membrane-associated proteins detected, 564 contained ≥1 unique peptide for quantification (Supplementary Data S9). Strikingly, proTGFβ1 was the only protein significantly enriched [log_2_ fold change (FC) ≥6] in ADAMTSL4-CT samples relative to controls, alongside ADAMTSL4 itself (Fig. 5A and B; Supplementary Table S3). Enrichment of the TRFE receptor (TFR1) in TRFE-treated cells confirmed assay specificity (Fig. 5B). These results identify proTGFβ1 as the top candidate surface interactor associated with ADAMTSL4-CT under these conditions.

ProTGFβ1 comprises the latency-associated peptide (LAP) and mature TGFβ1 within a single precursor. After dimerization and Furin-mediated processing in the endoplasmic reticulum/Golgi, the LAP dimer remains noncovalently bound to mature TGFβ1, forming the small latent complex (SLC) that sequesters TGFβ1 and prevents receptor engagement until activation (40, 41).

To determine whether ADAMTSL4-CT engages LAP or mature TGFβ1, we coexpressed V5- and 6×His-tagged ADAMTSL4-CT with full-length proTGFβ1 in HEK293T cells using constructs in which LAP and TGFβ1 were selectively tagged with HA and FLAG epitopes, respectively (Supplementary Fig. S5A). Intracellular processing by endogenous furin-like convertases produced discrete HA-LAP and FLAG-TGFβ1 species, as confirmed by Western blot analysis of input lysates (Fig. 5C and D; Supplementary Fig. S5B and S5C). Immunoprecipitation (IP) of HA-LAP with anti-HA antibody followed by anti-His immunoblotting revealed a specific association between ADAMTSL4-CT and LAP under the conditions tested (Fig. 5C). Conversely, no association was detected when TGFβ1 was immunoprecipitated using an anti-FLAG antibody (Fig. 5D). These results were corroborated by reciprocal IP of ADAMTSL4-CT using anti-V5 or anti-His antibodies, which co-precipitated HA-LAP but not FLAG-TGFβ1 (Supplementary Fig. S5B and S5C). Together, these findings support LAP as the relevant interacting component of the latent TGFβ1 complex engaged by ADAMTSL4-CT, although the precise residue-level binding interface remains undefined.

To independently support functional engagement between ADAMTSL4-CT and LAP, we used the NanoBiT luciferase complementation assay, which enables quantitative detection of interaction-compatible protein proximity. We fused LgBiT to LAP and SmBiT to ADAMTSL4-CT, as illustrated schematically (Fig. 5E), and transfected HEK293T cells with individual constructs or both constructs in combination. Coexpression of both fusion proteins resulted in a robust and quantitative luminescent signal in live cells, indicating specific interaction-compatible proximity between ADAMTSL4-CT and LAP (Fig. 5F). Consistent results were obtained in lysate-based NanoBiT assays using matched protein inputs (Supplementary Fig. S5D), further supporting functional engagement in a context independent of live-cell membrane organization.

### ADAMTSL4 Promotes Release of TGFβ1 from the Latent TGFβ1 Complex

We hypothesized that engagement of LAP by ADAMTSL4-CT could destabilize the latent LAP–TGFβ1 complex and thereby promote release of active TGFβ1, consistent with its shared thrombospondin type 1 repeat (TSR) features with thrombospondin-1, a known regulator of latent TGFβ activation (42). To test this, we adapted the NanoBiT system to monitor dissociation of the complex by tagging LAP with LgBiT and TGFβ1 with SmBiT within the full-length proTGFβ1 construct, as illustrated (Fig. 5G). In this configuration, luminescence reflects proximity between LAP and TGFβ1, and disruption of the complex leads to signal loss. Coexpression of V5-tagged ADAMTSL4-CT significantly reduced luminescence in cell lysates (Fig. 5H). Similarly, reduced luminescence was observed in live cells expressing NanoBiT-proTGFβ1 following treatment with recombinant ADAMTSL4-CT (Fig. 5I), supporting functional destabilization of the LAP–TGFβ1 complex.

To validate this finding in a physiologically relevant system, we treated differentiated C2C12 myotubes with recombinant ADAMTSL4-CT and measured free, active TGFβ1 levels in the culture supernatant by ELISA. ADAMTSL4-CT increased the amount of free TGFβ1 under basal conditions, and this effect was further amplified by HCl treatment, which is commonly used as an experimental condition to promote latent complex activation by weakening the LAP–TGFβ1 interaction (Fig. 5J). Together, these data support a model in which ADAMTSL4 engagement of LAP facilitates the liberation of mature TGFβ1.

### ADAMTSL4 Promotes Skeletal Muscle Wasting via TGFβ1−TβRI Signaling

To test whether proTGFβ1 is required for ADAMTSL4-induced myotube atrophy, we knocked down proTgfb1 in fully differentiated C2C12 myotubes using a lentivirus expressing a proTgfb1-targeted shRNA. Knockdown effectively reduced *Tgfb1* mRNA under both basal conditions and following treatment with rhADAMTSL4-CT (Fig. 5K). The rhADAMTSL4-CT-induced upregulation of *Tgfb1* (Fig. 5K) and *Ctgf* (Supplementary Fig. S5E), both TGFβ1–responsive genes, was significantly attenuated upon TGFβ1 knockdown, indicating that exogenous ADAMTSL4 activates autocrine TGFβ1 signaling. Importantly, the rhADAMTSL4-CT-induced increase in *Fbxo32* mRNA expression (Fig. 5L) as well as myotube atrophy (Fig. 5M; Supplementary Fig. S5F), as assessed by immunofluorescence-based myotube diameter analysis, were abolished under TGFβ1 knockdown conditions, supporting a model in which ADAMTSL4, via LAP engagement, regulates local active TGFβ1 levels to mediate its catabolic effects.

To further directly test whether ADAMTSL4-induced signaling depends on TGFβ1 activity, we performed functional neutralization experiments using the TGFβ–blocking monoclonal antibody 1D11. Preincubation of C2C12 myotubes with 1D11 effectively abrogated rhADAMTSL4-CT–induced SMAD2 phosphorylation, as assessed by Western blot analysis and corresponding quantification (Fig. 5N; Supplementary Fig. S5G). Consistently, 1D11 treatment suppressed the induction of canonical TGFβ target genes (*Tgfb1* and *Ctgf*; Fig. 5O and P) and atrophy-related genes (*Fbxo32* and *Map1lc3*; Fig. 5Q and R) triggered by ADAMTSL4, demonstrating that ADAMTSL4-driven downstream responses are dependent on TGFβ activity.

To further test whether ADAMTSL4-induced responses depend on LAP engagement, C2C12 myotubes were preincubated with recombinant human LAP (rhLAP) prior to rhADAMTSL4-CT treatment. The rhADAMTSL4-CT–induced SMAD2 phosphorylation (Supplementary Fig. S5H and S5I), *Fbxo32* and *Map1lc3* upregulation (Supplementary Fig. S5J and S5K), and myotube diameter reduction (Supplementary Fig. S5L and S5M) were all abolished in the presence of rhLAP, consistent with sequestration of exogenous rhADAMTSL4. This functional rescue supports the hypothesis that ADAMTSL4 releases active TGFβ1 via LAP engagement, consistent with previous biochemical assays. Notably, direct treatment with recombinant TGFβ1 recapitulated the effects of ADAMTSL4, inducing SMAD2 phosphorylation, upregulating *Tgfb1*, *Pai1*, and *Fbxo32* mRNA levels (Supplementary Fig. S5N–S5Q), confirming that TGFβ1 is sufficient to trigger the catabolic program observed with ADAMTSL4 treatment, consistent with prior reports in cancer cachexia (9).

Finally, to assess whether TGFβ receptor signaling mediates ADAMTSL4-induced atrophy, C2C12 myotubes were treated with rhADAMTSL4 in the presence or absence of small-molecule TβRI (ALK5) inhibitors. The robust induction in SMAD2 phosphorylation (Fig. 5S; Supplementary Fig. S5R) and *Tgfb1* (Supplementary Fig. S5S) and *Ctgf* mRNA expression (Fig. 5T) in ADAMTSL4-treated myotubes was strongly suppressed in the presence of three different TβRI inhibitors. Furthermore, the upregulation of *Fbxo32* mRNA (Fig. 5U) and C2C12 myotube atrophy (Fig. 5V and W) induced by ADAMTSL4 were significantly attenuated when cells were pretreated with different TβRI inhibitors. Importantly, these findings support functional LAP engagement and latent complex destabilization by ADAMTSL4 but do not define the residue-level interface or precise structural mechanism by which ADAMTSL4 promotes activation of latent TGFβ1. In summary, these findings support a model in which ADAMTSL4 promotes local activation of latent TGFβ1 through LAP engagement, thereby triggering TβRI-dependent signaling and catabolic responses that culminate in muscle atrophy (Fig. 5X).

### ADAMTSL4 Promotes Adipocyte Wasting through TβRI Signaling

Given that ADAMTSL4 locally activates TGFβ to induce skeletal muscle atrophy, we next tested whether TβRI mediates ADAMTSL4-induced wasting in differentiated murine 3T3-L1 adipocytes. rhADAMTSL4-CT robustly induced lipolysis and SMAD2 phosphorylation, effects that were effectively abolished by all three TβRI inhibitors tested (Supplementary Fig. S6A–S6C). To extend these findings to a human context, we used fully differentiated adipocytes derived from hTERT-immortalized human mesenchymal stem/stromal cells. As in murine adipocytes, rhADAMTSL4-CT–induced lipolysis and SMAD2 activation were strongly inhibited by TβRI cotreatment (Supplementary Fig. S6D–S6F).

Given the consistent TβRI-dependent wasting observed *in vitro* and *in vivo*, we asked whether ADAMTSL4 also activates TGFβ–responsive genes in white adipose tissue of C26 tumor-bearing mice. Targeted gene set enrichment analysis (GSEA) on RNA-seq data from eWAT and iWAT revealed significant suppression of noncanonical TGF-responsive genes in eWAT (Supplementary Fig. S6G and S6H) and canonical TGF-responsive genes in iWAT (Supplementary Fig. S6I and S6J) from mice bearing C26-shAdamtsl4 tumors compared with controls. Notably, top upregulated genes within the ALK pathway identified by GSEA corresponded to key TGFβ signaling components, reinforcing that ADAMTSL4-driven adipocyte wasting operates via TGFβ signaling.

Collectively, these *in vitro* and *in vivo* findings across murine and human adipocytes support the conclusion that tumor-derived ADAMTSL4 promotes adipocyte wasting through local activation of TGFβ and TβRI-dependent signaling.

### Tumor-Derived ADAMTSL4 Drives TGFβ–Dependent Muscle Fibrosis

**Figure 6.**
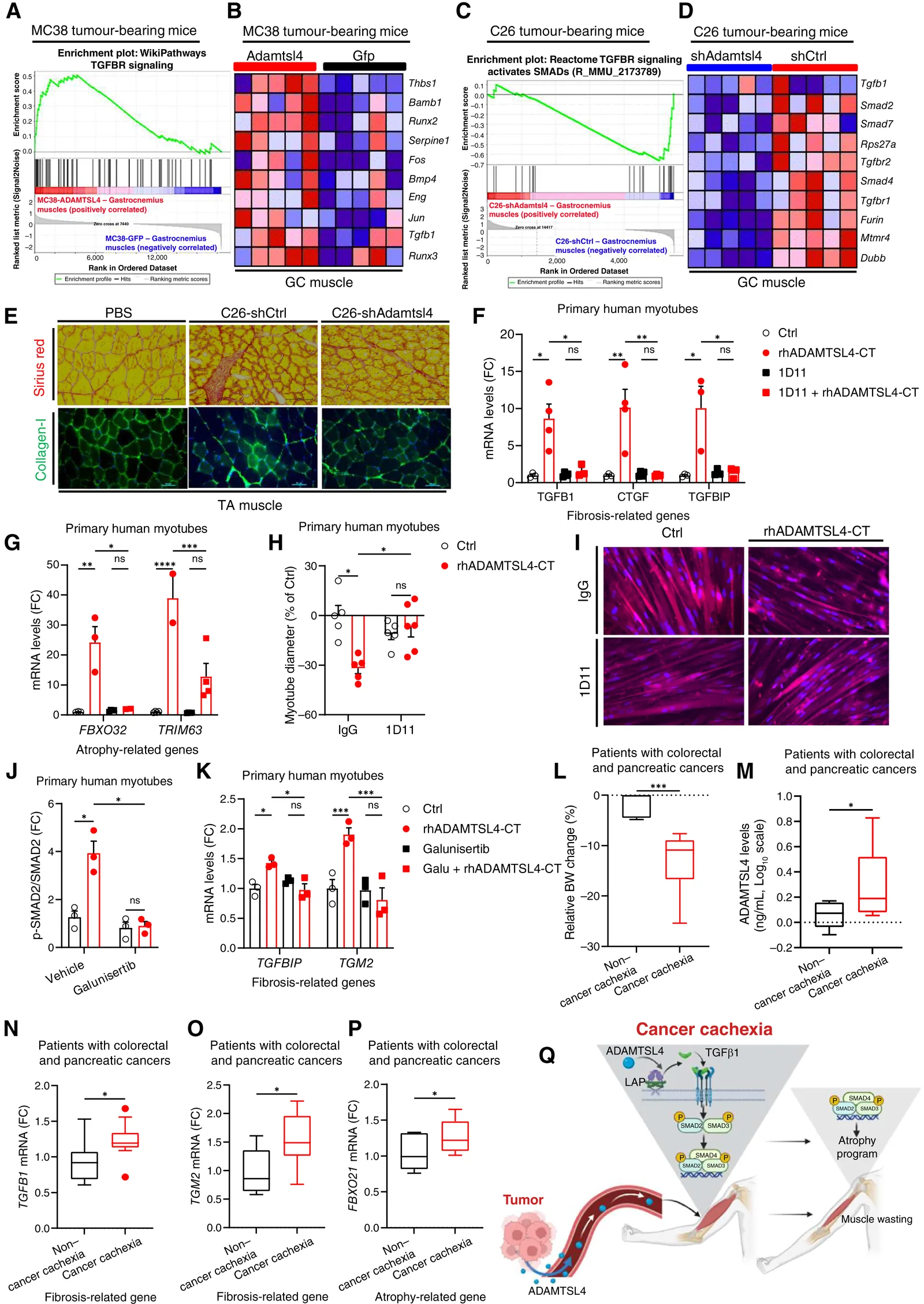
ADAMTSL4 induces skeletal muscle fibrosis via local TGFβ signaling and activates fibrosis-related genes in human myotubes and muscles of patients with cancer cachexia. **A** and **B**, GSEA (**A**) of GC muscle from MC38-GFP vs. MC38-ADAMTSL4 tumor-bearing mice showing enrichment of the “TGFβ receptor signaling” pathway and heatmap (**B**) of the top 10 DEGs within this pathway. **C** and **D**, GSEA (**C**) of GC muscle from C26-shCtrl vs. C26-shAdamtsl4 tumor-bearing mice showing enrichment of “TGFβ receptor signaling activates SMADs” and heatmap (**D**) of the top 10 DEGs. **E,** Representative Sirius Red (top) and collagen-I (bottom) staining in GC muscle sections from PBS-injected, C26-shCtrl, and C26-shAdamtsl4 tumor-bearing mice (scale bar, 100 μm). **F–K,** Human primary myotubes were treated with rhADAMTSL4-CT (250 ng/mL) in the presence or absence of TGFβ pathway blockade: (**F**) mRNA levels of fibrosis-related genes (*TGFB1*, *CTGF*, and *TGFBI*); (**G**) atrophy-related genes (*FBXO32* and *TRIM63*) measured by qPCR; (**H**) quantification of myotube diameter; (**I**) representative images of myotubes treated with vehicle or rhADAMTSL4-CT ± TGFβ1–neutralizing antibody 1D11 (1.5 μg/mL; scale bar, 100 μm); (**J**) Western blot analysis of phosphorylated SMAD2 (p-SMAD2); (**K**) qPCR analysis of CTGF and TGM2 mRNA levels in myotubes treated with vehicle or rhADAMTSL4-CT ± galunisertib (10 μmol/L), a TGFBR1 inhibitor. **L** and **M,** Relative body weight change and circulating ADAMTSL4 levels measured by ELISA in an exploratory human cancer subcohort including patients with colorectal cancer and PDAC classified as cachectic (cancer cachexia, *n* = 15) or noncachectic (non–cancer cachexia, *n* = 7). **N–P,** qPCR analysis of *TGFB1*, *TGM2*, and *FBXO21* mRNA expression in rectus abdominis muscle biopsies from non–cancer cachexia patients and those with cancer cachexia with colorectal cancer/PDAC. **Q,** Schematic model depicting how tumor-derived ADAMTSL4 promotes muscle wasting via local activation of TGFβ signaling through LAP engagement and TGFBR1-dependent transcriptional responses. Data are shown as the mean ± SEM unless indicated otherwise. Each dot represents one biological replicate. Statistical analysis: weighted Kolmogorov–Smirnov test for GSEA enrichment scores (**A** and **C**); two-way mixed-effects model with Tukey or Holm–Šídák multiple-comparisons test (**F–H**, **J**, and **K**); for circulating ADAMTSL4 measurements in human plasma (**M**), ELISA values were log_10_-transformed and analyzed using a two-tailed Mann–Whitney test; human gene expression comparisons (**N–P**) were analyzed using two-tailed Mann–Whitney tests. Significance is denoted as *, *P* < 0.05; **, *P* < 0.01; ***, *P* < 0.001; ****, *P* < 0.0001; ns, not significant. [**Q,** Created in BioRender. Machado, J. (2026) https://BioRender.com/4sidioq.]

To assess whether tumor-derived ADAMTSL4 activates TGFβ signaling *in vivo*, we performed targeted GSEA on skeletal muscle RNA-seq data from reciprocal cachexia models: MC38 tumors overexpressing *Adamtsl4* and C26 tumors with *Adamtsl4* suppression. TGFβ receptor signaling was significantly enriched in GC muscles of MC38-ADAMTSL4 mice and conversely suppressed in muscles of C26-shAdamtsl4 mice compared when compared with their respective controls (Fig. 6A–D). These transcriptional changes coincided with preserved muscle mass, mirroring our *in vitro* observations, and support a model in which tumor-derived ADAMTSL4 enhances local TGFβ activity in muscle.

Consistent with the known effects of TGFβ in tissue fibrosis, C26 tumor-bearing mice displayed increased collagen deposition in skeletal muscle, as assessed by Sirius Red and collagen-I staining (Fig. 6E). *Adamtsl4* knockdown markedly attenuated this fibrotic phenotype and reduced expression of TGFβ target genes (Fig. 6C–E). Notably, fibrosis- and TGF-related transcripts were also elevated in the hearts of cachectic C26 mice (Supplementary Fig. S6K), indicating that tumor-derived ADAMTSL4 may drive multiorgan remodeling.

Circulating total TGFβ levels were unchanged across genotypes (Supplementary Fig. S6L), consistent with a model in which ADAMTSL4 enhances tissue-level activation of latent TGFβ without measurably altering systemic total TGFβ levels. This is further supported by observations in patients with cachectic colorectal cancer, in whom circulating total TGFβ levels were not significantly different between noncachectic and cachectic groups (Supplementary Fig. S1P). This defines a previously unrecognized tumor-host axis, whereby a tumor-secreted protein orchestrates local tissue remodeling without altering circulating cytokine levels.

### ADAMTSL4-TGFβ Signaling Drives Muscle Fibrosis in Patients with Cancer Cachexia

To translate our findings to humans, we used primary human skeletal muscle cells (HSkMC) from healthy donors to test whether ADAMTSL4 similarly drives wasting via TGFβ signaling. Treatment with rhADAMTSL4-CT robustly induced TGFβ target genes (*CTGF* and *TGFBPI*) and atrophy genes (*FBXO32* and *TRIM63*), effects that were markedly blunted by the TGFβ–neutralizing antibody 1D11 (Fig. 6F and G). Likewise, rhADAMTSL4-CT-induced myotube atrophy was fully prevented by 1D11 (Fig. 6H and I). Inhibition of TβRI/ALK5 with galunisertib, a clinically tested small-molecule inhibitor, similarly blocked SMAD2 phosphorylation and induction of fibrosis genes (*TGFBPI* and *TGM2*) in human myotubes (Fig. 6J and K), demonstrating translational relevance of the ADAMTSL4–TGFβ axis.

We next examined skeletal muscle samples obtained intraoperatively during tumor resection from a small, paired cohort of patients with cancer with matched plasma samples, enabling translational assessment of the ADAMTSL4–TGFβ axis. This cohort comprised colorectal cancer cases together with a limited number of pancreatic cancer samples, included to evaluate the consistency of the mechanism across gastrointestinal tumor contexts while prioritizing paired tissue-circulation relationships. Given the modest sample size and cohort heterogeneity, circulating ADAMTSL4 values are shown on a log_10_ scale, and group comparisons were analyzed using two-tailed Mann–Whitney tests, providing a distribution-aware framework for this exploratory matched cohort. Cachectic patients exhibited elevated circulating ADAMTSL4 relative to noncachectic controls (Fig. 6L and M). Critically, atrophic muscles from these patients displayed increased expression of fibrosis-related genes (*TGFB1* and *TGM2*) and the atrophy mediator *FBXO21* (Fig. 6N–P; ref. 43), whereas plasma TGFβ1 levels were unchanged (Supplementary Fig. S6L), supporting a model in which circulating ADAMTSL4 drives local TGFβ activation in skeletal muscle in humans. Notably, despite the limited size of this matched cohort, the coordinated increase in circulating ADAMTSL4 together with muscle expression of TGFB1, TGM2, and FBXO21 supports a conserved ADAMTSL4–TGFβ–atrophy axis in human cancer cachexia.

Collectively, these results establish the ADAMTSL4–TGFβ axis as a tumor–host cross-talk mechanism mediating muscle wasting and fibrosis in cancer cachexia and identify ADAMTSL4 as a potential therapeutic target to preserve musculoskeletal integrity by inhibiting local TGFβ activation (Fig. 6Q).

## Discussion

Cancer cachexia is a multifactorial syndrome characterized by systemic metabolic dysfunction and wasting of skeletal muscle and adipose tissue (6, 8, 9). Tumor-derived factors are widely recognized as primary drivers of cancer cachexia. In this study, we identify ADAMTSL4 as a previously uncharacterized tumor-secreted mediator of cancer cachexia that promotes tissue wasting by enhancing local activation of latent TGFβ signaling. Our study provides three principal insights: (i) Circulating ADAMTSL4 levels are elevated in cancer cachexia across preclinical models and patient cohorts; (ii) tumor-derived ADAMTSL4 is both necessary and sufficient to drive coordinated skeletal muscle and white adipose tissue wasting *in vivo*; and (iii) ADAMTSL4 engages the LAP of TGFβ1, promoting local release of active TGFβ and triggering SMAD-dependent catabolic programs. Importantly, our data support a local activation model in which ADAMTSL4 enhances TGFβ activity in target tissues without necessarily altering circulating total TGFβ levels, which predominantly reflect latent pools and are assay-dependent.

Elevated circulating ADAMTSL4 has been reported in patients with gastric cancer (44) and spinal muscular atrophy (45), suggesting that ADAMTSL4 may represent a broader feature of wasting syndromes (46). Our gain- and loss-of-function tumor models indicate that tumor-derived ADAMTSL4 is a major driver of tissue wasting. Mechanistically, LAP–TGFβ1 was identified as a prominent extracellular interactor of ADAMTSL4 in differentiated myotubes using LRC-TriCEPS and mass spectrometry. Inhibition of TGFβ1 or TβRI fully blocked ADAMTSL4-induced wasting in muscle and adipocytes, supporting a causal requirement for TGFβ1−TβRI signaling in ADAMTSL4-driven wasting. Although our biochemical and NanoBiT assays support functional LAP engagement and destabilization of the latent complex, we have not defined the residue-level binding interface or performed structure-guided mutagenesis to map the molecular determinants of interaction. Therefore, although our data demonstrate LAP-dependent activation of TGFβ signaling, the precise structural mechanism by which ADAMTSL4 promotes latent complex destabilization remains to be determined. These findings complement prior studies linking TGFβ release from metastatic bone lesions to muscle weakness (9) and position ADAMTSL4 as a previously unrecognized upstream mediator of TGFβ–driven wasting in cancer cachexia.

ADAMTSL4 contains multiple TSRs. TSR-containing thrombospondin-1 (TSP1) is a well-established activator of latent TGFβ through LAP engagement (42), raising the possibility that TSR modules within ADAMTSL4 may contribute to a mechanistically analogous, nonproteolytic mode of latent complex destabilization. Consistent with this concept, our biochemical and NanoBiT data support LAP engagement by ADAMTSL4 and functional destabilization of the SLC; however, the specific TSR(s) involved and the precise mode of destabilization remain to be defined. Activated TGFβ induces canonical SMAD2/3 transcription of atrophy genes in muscle and lipolytic genes in adipose tissue (36, 43, 47, 48) and engages noncanonical pathways, including FOXO3A and STAT3, synergizing with SMADs to enhance proteasomal activity and autophagy (49–51). ADAMTSL4 thus triggers a broad catabolic program across cachectic tissues (52), validated in primary human myotubes and adipocytes via TGFβ−TβRI-dependent mechanisms (53, 54).

Beyond atrophy, TGFβ drives tissue fibrosis (55). Consistent with prior reports (56–58), we observed increased fibrotic gene expression and collagen deposition in skeletal muscle of cachectic mice and patients. Suppressing tumor-derived ADAMTSL4 attenuated both atrophy and fibrosis *in vivo*, correlating with reduced activation of TGFβ–responsive genes in muscle, adipose tissue, and heart. Fibrotic signatures were recapitulated in human myotubes and patient biopsies, underscoring translational relevance (56–60). Furthermore, our data support ADAMTSL4 as a previously unrecognized tumor-derived modulator of latent TGFβ activation via LAP engagement, with potential implications beyond cancer cachexia.

Targeting TGFβ signaling shows promise in preclinical cancer and cachexia models (61, 62), but systemic blockade is limited by pleiotropic roles in tissue homeostasis. In contrast, ADAMTSL4 inhibition offers a more selective strategy. Tumor ADAMTSL4 suppression did not affect proliferation *in vitro* or tumor burden *in vivo*, supporting its role as a cachexia-specific mediator. Clinically, elevated circulating ADAMTSL4 correlated with cachexia severity across cohorts (ELISA concentrations and Olink NPX, as indicated) and was associated with reduced OS but not disease-free survival, resembling patterns observed for GDF15 (14, 29).

Importantly, in both gain- and loss-of-function tumor-bearing mouse models, progressive loss of lean and fat mass occurred despite preserved food intake. Because food intake was assessed at the cage level, subtle differences in caloric intake cannot be fully excluded. Nevertheless, this phenotype is not consistent with anorexia-driven weight loss (12) but rather with a net negative energy balance. Although indirect calorimetry was not performed in the current study, the coordinated depletion of skeletal muscle and adipose tissue together with activation of proteolytic and lipolytic gene programs is consistent with increased substrate utilization and altered metabolic efficiency (63). TGFβ signaling has been linked to enhanced muscle protein turnover, activation of ubiquitin–proteasome and autophagy pathways, and increased adipose tissue lipolysis, all of which may contribute to energy imbalance (9, 48, 49, 52–54, 59). Future studies using metabolic cage analysis will be required to directly quantify energy expenditure and substrate oxidation in this context.

In epithelial cancers, TGFβ is tumor-suppressive early, but in advanced stages, prevalent in cachexia, it promotes epithelial–mescenchymal transition (64), metastasis (65), and immunosuppression (66). TGFβ also induces activin secretion in colon cancer cell lines (67), suggesting that ADAMTSL4 may indirectly amplify other TGFβ superfamily members via SMAD2/3 feedforward loops (36, 68). Thus, targeting ADAMTSL4 could prevent tissue wasting while enhancing antitumor immunity, without disrupting tumor-suppressive functions induced by TGFβ.

Several considerations remain. Although our models identify tumor cell–intrinsic ADAMTSL4 as a major source driving cachexia, potential contributions from stromal or host-derived ADAMTSL4, particularly in late-stage cachexia, require further study. Structural insights into the ADAMTSL4–LAP interface, including residue-level mapping and interface-disrupting mutants, will be required to define the molecular determinants of binding and enable rational therapeutic targeting of this interaction. Although ADAMTSL4 may represent a more selective upstream modulator of TGFβ activation, the long-term consequences of systemic ADAMTSL4 inhibition will need to be carefully evaluated, given the pleiotropic roles of TGFβ in tissue homeostasis, repair, and immunity. Finally, the observed heterogeneity in circulating ADAMTSL4 levels among patients highlights the need for stratification strategies to identify those most likely to benefit from targeted intervention. Importantly, although effect sizes in human cohorts are inherently modest due to interpatient heterogeneity, the consistency and coordinated regulation of multiple pathway components across matched samples provide strong support for the biological relevance of this axis.

In summary, our findings identify ADAMTSL4 as a tumor-derived upstream regulator of local TGFβ activation in skeletal muscle and adipose tissue that links tumor burden to systemic wasting and highlights a potential therapeutic target to counteract cancer cachexia (Fig. 6Q).

## Methods

### Cell Lines

The murine colon cancer cell lines MC38 (cat. #SCC172; RRID: CVCL_B288), C26 (German Cancer Research Center), and LLC (cat. #CRL-1642; RRID: CVCL_4358), as well as murine PDAC cell lines derived from KPC (*Kras*^LSL-G12D^;*Trp53*^LSL-R172H^;*Pdx1-Cre*) mice (a kind gift from Dieter Saur, Technical University of Munichy), were cultured in high-glucose DMEM with pyruvate (Life Technologies, #41966052) supplemented with 10% fetal bovine serum (FBS, Sigma-Aldrich, #F7524) and 1% penicillin–streptomycin (Thermo Fisher Scientific, #15140122). For *in vivo* inoculation, cells were harvested at ∼80% confluency, trypsinized, counted, and resuspended in Dulbecco’s PBS (DPBS; Thermo Fisher Scientific, #14190250).

Murine C2C12 myoblasts (ATCC, cat. #CRL-1772; RRID: CVCL_0188) were cultured in growth medium (high-glucose DMEM with pyruvate, 10% FBS, and 1% penicillin–streptomycin) until 80% to 100% confluency. Myotube differentiation was induced by switching to medium containing 2% horse serum for 5 days.

HSkMCs (ATCC, cat. #PCS-950-010) were cultured in Mesenchymal Stem Cell Basal Medium (ATCC, #PCS-500-030) supplemented with the Primary Skeletal Muscle Cell Growth Kit (ATCC, #PCS-950-040) and 1% penicillin–streptomycin, following the manufacturer’s instructions. Differentiation was induced using the Skeletal Muscle Differentiation Tool (ATCC, #PCS-950-050), with media refreshed every 2 days. Experiments were conducted after 5 days of differentiation.

3T3-L1 preadipocytes (ATCC, cat. #CL-173; RRID: CVCL_0123) were cultured in high-glucose DMEM with pyruvate, supplemented with 10% FBS and 1% penicillin–streptomycin. Upon reaching full confluency (day 0), differentiation was induced by adding 1 μg/mL insulin (Sigma, #I9278), 0.25 μmol/L dexamethasone (Sigma, #D4902), 0.5 mmol/L IBMX (Sigma, #I7018), and 2 μmol/L rosiglitazone (Sigma, #R2408). After 2 days, dexamethasone, IBMX, and rosiglitazone were removed; insulin was withdrawn after 4 days. Adipocytes were cultured for an additional 4 days prior to experimentation.

Telomerase-immortalized human mesenchymal stem cells (hMSC-Tert) were maintained in high-glucose DMEM with pyruvate, 10% FBS, and 1% penicillin–streptomycin. Two days after reaching confluency (day 0), adipogenic differentiation was induced with medium containing 5% FBS, 2 μmol/L insulin, 0.1 μmol/L dexamethasone, 0.5 mmol/L IBMX, 1 μmol/L rosiglitazone (Cayman Chemical), and 1 μmol/L U0126 (Calbiochem). Media were refreshed every 2 to 3 days. Rosiglitazone was removed on day 12, and the remaining additives were withdrawn on day 19.

HEK293T cells (ATCC, #CRL-3216; RRID: CVCL_0063) were cultured in high-glucose DMEM supplemented with 10% FBS (Sigma-Aldrich, #F7524) and 1% penicillin–streptomycin (Thermo Fisher Scientific, #15140122). Cells were used for lentiviral production and as a heterologous expression system to validate ADAMTSL4 secretion and functional sufficiency.

All cell lines were routinely tested for *Mycoplasma* contamination by PCR (Promokine, #PK-CA91-1048).

### Lentivirus Production and Transduction

For lentivirus production, HEK293T cells (∼70% confluency) were transfected with third-generation packaging plasmids and lentiviral transfer vectors using Lipofectamine 2000 (Thermo Fisher Scientific, #11668019) in Opti-MEM (Life Technologies, #31985062), according to the manufacturer’s instructions. Transfer plasmids encoded GFP, ADAMTSL4, a nontargeting control shRNA (shCtrl), or shRNA targeting ADAMTSL4 (shAdamtsl4), together with packaging (psPax2, Addgene, #12260) and envelope (pMD2.G, Addgene, #12259; a gift from Didier Trono) plasmids. After 24 hours, the medium was replaced with fresh medium containing 2% fatty acid–free bovine serum albumin (BSA; Sigma-Aldrich, #A7030). Viral supernatants were collected at 48 and 72 hours after transfection, filtered through a 0.45-μm membrane, and either used immediately or stored at −80°C.

To generate cells with stable overexpression of ADAMTSL4, non–cachexia-inducing MC38 cells (∼50% confluent) were transduced with lentivirus encoding either GFP or ADAMTSL4 in the presence of 10 μg/mL Polybrene (Santa Cruz, #sc-134220) for 24 hours and selected with 500 μg/mL hygromycin (Thermo Fisher Scientific, #10687010) for 3 days. To generate cells with stable knockdown of ADAMTSL4, cachexia-inducing C26 or 8025 cells were transduced similarly with shCtrl or shADAMTSL4 lentivirus in the presence of 10 μg/mL Polybrene for 24 hours and selected with 3.5 μmol/L puromycin (Thermo Fisher Scientific, #A1113803) for 3 days.

### Assessment of Cachexia Phenotypes *In Vitro*

To assess cachexia *in vitro* in adipocytes, fully differentiated 3T3-L1 or hMSC-Tert adipocytes were incubated for 24 hours with cancer cell–conditioned medium or with the indicated compounds (as described in the “Results” section). Cachexia was evaluated by quantifying glycerol release into the medium using a commercial kit (Sigma-Aldrich, #F6428), normalized to total protein content per well. Additional experiments were performed to assess protein abundance and phosphorylation status under different conditions.

To assess cachexia *in vitro* in muscle cells, murine and human myotubes were fixed in 4% paraformaldehyde for 10 minutes and stained with MitoTracker Green FM (Thermo Fisher Scientific, #M7514), MitoTracker Red FM (Thermo Fisher Scientific, #M22425), and DAPI. Approximately 10 images per well were acquired using a 40× objective (Nikon Eclipse Ts2). Roughly 100 myotubes per well were quantified using Fiji/ImageJ (Fiji, RRID: SCR_002285; ImageJ, RRID: SCR_003070). Additional assays were performed to assess gene expression, protein/phospho-protein levels, and secreted TGFB1, as described in the “Results” section.

### Preclinical Models of Cancer Cachexia

All animal experiments were approved by the institutional animal welfare officer and conducted under licenses issued by the Government of Upper Bavaria (ROB-55.2-2532.Vet_02-16-136 and ROB-55.2-2532.Vet_02-18-93). Male BALB/c (Charles River Laboratories; strain code: 028; RRID: IMSR_CRL:028) or C57BL/6N mice (8–12 weeks old; Charles River Laboratories; strain code: 027; RRID: IMSR_CRL:027) were housed in specific pathogen-free conditions with a 12-hour light/dark cycle at 22°C and *ad libitum* access to standard chow (Kliba Nafag, #3437, Promivi Kliba AG) and water. Animals were randomly assigned to groups with equal distribution of body weight, lean mass, and fat mass (confirmed by nonsignificant statistical analysis).

To assess the association between circulating ADAMTSL4 and cancer cachexia, mice were subcutaneously injected in the right flank with 100 μL of DPBS (Thermo Fisher Scientific, #14190250) containing either 1 × 10^6^ C26 colon carcinoma cells (BALB/c), 2 × 10^6^ LLC cells (C57BL/6J), or 1.5 × 10^6^ MC38 colon adenocarcinoma cells (C57BL/6N), a non–cachexia-inducing control. Nontumor controls received 100 μL of DPBS alone. Animals were monitored daily for body weight, food intake, and tumor growth over 2 to 3 weeks.

We also used the genetically engineered C57BL/6J-APC^Min/+^ mouse model (The Jackson Laboratory; stock #002020; RRID: IMSR_JAX:002020), which spontaneously develops intestinal polyps mimicking human colorectal cancer and is a well-established model of spontaneous cachexia.

To evaluate whether tumor-derived ADAMTSL4 is sufficient to induce cachexia, MC38 cells were stably transduced to overexpress Adamtsl4 (MC38-ADAMTSL4) or GFP control (MC38-GFP) via lentiviral transduction. Mice were injected subcutaneously with 1.5 × 10^6^ cells in 100 μL DPBS and monitored for cachexia progression.

To test whether Adamtsl4 is required for cachexia development, C26 cells were engineered to stably express a shRNA targeting Adamtsl4 (C26-shAdamtsl4) or a nontargeting control (C26-shCtrl). Male BALB/c mice (12 weeks old) received 1.5 × 10^6^ cells subcutaneously or DPBS as a control. Mice were sacrificed upon 10% to 15% body weight loss or when tumors reached humane endpoints (ulceration or >15 mm). Tumor size was matched between groups at endpoint.

For cachexia-related mortality, mice bearing C26-shAdamtsl4 or control tumors were monitored longitudinally and euthanized upon reaching refractory cachexia (15%–20% body weight loss). The experiment was terminated 30 days later, when tumors reached humane endpoints (ulceration or >15 mm) as per Helmholtz Center Munich ethical guidelines.

In gain- and loss-of-function tumor models of cancer cachexia, food intake was measured daily by weighing chow pellets in each cage at the same time each day. Mice were housed two per cage, and total food consumption per cage was calculated as the difference between the amount of food provided and the amount remaining on the following day. Food spillage was visually inspected and excluded from bedding prior to weighing whenever possible. Daily intake was normalized to the number of mice per cage and expressed as grams per mouse per day. When one mouse was removed from a cage due to endpoint criteria, intake was normalized to the remaining animal. Group sizes and variance are indicated in the corresponding figure legends.

At sacrifice, blood was collected using EDTA-coated syringes and centrifuged at 500 × *g* for 10 minutes at 4°C to isolate plasma, which was snap-frozen in liquid nitrogen and stored at −80°C. Tissues including tumor, liver, heart, spleen, iWAT, eWAT, interscapular BAT, TA, and GC muscles were dissected, snap-frozen, and processed for downstream analysis. Body composition was assessed using EchoMRI (Echo Medical Systems, Houston), and plasma nonesterified fatty acids were measured using a Beckman Coulter AU480 Chemistry Analyzer.

### Patient Cohorts and Samples

This study included independent human cancer cachexia cohorts, each approved by the corresponding institutional review boards. Written informed consent was obtained from all participants prior to inclusion in accordance with the Declaration of Helsinki. Plasma or serum samples were used to assess the association between circulating ADAMTSL4 levels and cancer cachexia. A Key Resources Table with further information is provided (Supplementary Tables S1 and S2).

Patients with colorectal cancer (including colon and rectal tumors; stages I–IV) were recruited from two independent clinical centers: (i) the Department of Surgery, Klinikum Rechts der Isar, Technical University of Munich (Germany), under the ethical approvals #1946/07 and #409/16S; and (ii) the Surgical Clinic of the University Hospital of São Paulo (Brazil), approved by the Ethics Committee on Research Involving Human Subjects of the University of São Paulo Biomedical Sciences Institute (CEP 1151/13, CAAE 5493116.6.0000467) and the University Hospital (CEP 1390/14, CAAE 54930116.6.3001.0076). Written informed consent was obtained from all participants prior to inclusion, in accordance with the Declaration of Helsinki. Patient inclusion was based on histologic confirmation of colorectal cancer. Cancer staging followed the eighth edition of the Union for International Cancer Control tumor–node–metastasis classification system. Body weight was recorded at hospital admission, and weight history for the preceding 6 months was retrospectively assessed. Cachexia was defined according to the consensus criteria by Fearon and colleagues (25), and patients not meeting these criteria were classified as noncachectic. Blood samples were collected prior to surgery (Germany) or following overnight fasting before surgery (Brazil). Circulating biochemical parameters, including CRP, hemoglobin, and albumin, were measured using standard clinical chemistry platforms (Cobas 8000 system, Roche Diagnostics, or LABMAX 240 analyzer, Labtest) or obtained from hospital records. Plasma albumin was determined by the bromcresol green method as previously described Prokopchuk and colleagues (69). Data from both centers were analyzed as a combined colorectal cancer cohort.

A subset of patients recruited from the Department of Surgery, Klinikum Rechts der Isar, Technical University of Munich (Germany), including individuals with colorectal cancer and PDAC, was used for analyses involving matched plasma and skeletal muscle biopsies. Rectus abdominis muscle biopsies were obtained intraoperatively during tumor resection. In this subcohort, circulating ADAMTSL4 levels were measured in plasma, and gene expression analyses in skeletal muscle focused on *TGFB1*, *TGM2*, and *FBXO21* as representative components of the ADAMTSL4–TGFβ–atrophy axis. All procedures were conducted under the same ethical approvals (1946/07 and #409/16S).

NSCLC cohort (TRACERx): The TRACERx cohort is a UK-wide, prospective, multicentre observational study in patients with early-stage NSCLC designed to investigate tumor evolution through longitudinal and multiregional tumor sampling (ClinicalTrials.gov: NCT01888601). The study was approved by the London–Chelsea Research Ethics Committee (13/LO/1546), and written informed consent was obtained from all participants. Plasma proteomic profiling was conducted using the Olink Explore 3072 platform, as described in Al-Sawaf and colleagues (29). NPX values at the time of disease recurrence were compared between cachectic and noncachectic patients. NPX values represent log_2_-scaled relative protein abundance units and are not directly comparable in magnitude with absolute concentrations obtained by ELISA in other cohorts. Patients with new primary tumors during follow-up were excluded from analysis. Cachexia was defined as >5% weight loss from baseline to relapse or >2% weight loss in patients with baseline BMI <20 kg/m^2^. This cohort was further stratified by tumor histology into LUAD and lung squamous cell carcinoma subgroups to examine potential histotype-specific associations between circulating ADAMTSL4 and cachexia.

### Histology and Microscopy

TA muscles and hearts were embedded in optimal cutting temperature compound (Sakura Finetek) after freezing in liquid nitrogen–cooled isopentane. Cryosections of TA muscle were stained with WGA Alexa Fluor 488 Conjugate (Thermo Fisher Scientific, W11261) and DAPI and visualized using a Nikon Eclipse Ci Upright Microscope (20× magnification). For each mouse, CSA was quantified from >500 fibers across five distinct regions within the same anatomic area of the TA muscle using ImageJ/Fiji (version 1.51). For each mouse, representative images, mean CSA quantification, and fiber-size distribution plots were derived from the same TA muscle sections.

### ELISA

Protein levels of human and murine ADAMTSL4 (Cusabio; CSB-EL001320HU and EKC36258-BM, respectively), TGFβ1 (R&D Systems; DB100C), and GDF15 (R&D Systems; DGD150) were quantified using specific ELISA kits according to the manufacturers’ instructions. Absolute concentrations were calculated based on standard curves and are reported as ng/mL for ADAMTSL4 or pg/mL for TGFβ1 and GDF15. For statistical analyses of human ELISA-based biomarker data, values were additionally analyzed on a log_10_-transformed scale to account for right-skewed distributions, whereas absolute values are shown in the figures and reported for biological interpretation. Limits of detection (LOD) and quantification were defined according to the kit manufacturer’s specifications. Values below the LOD were handled according to the manufacturer’s guidelines and were not imputed; analyses were performed on available quantitative values. Olink-derived ADAMTSL4 measurements from the TRACERx cohort are reported separately as NPX values (log_2_-scaled relative abundance units) and were analyzed within that cohort only.

### Expression and Purification of Recombinant Proteins

Recombinant human ADAMTSL4 C-terminal fragment (ADAMTSL4-CT) containing a C-terminal V5 and 6×His tag was expressed using the Expi293 Expression System (Thermo Fisher Scientific, cat. #A14635). Expi293F cells (Thermo Fisher Scientific, cat. #A14527) were cultured in Expi293 Expression Medium (Thermo Fisher Scientific, #A1435101) in a humidified shaker incubator at 37°C with 8% CO_2_, rotating at 125 rpm. For recombinant protein production, Expi293F cells were transiently transfected with the ADAMTSL4-CT expression vector using the ExpiFectamine 293 reagent. Supernatants were harvested 3 days after transfection by sequential centrifugation (300 × *g* for 10 minutes, then 2,000 × *g* for 10 minutes, both at 4°C), followed by filtration (0.45 μm). ADAMTSL4-CT was purified using Capturem His-Tagged Purification Maxiprep Columns (Takara Bio, #635715 or #635719) according to the manufacturer’s instructions. Imidazole was removed from the eluate using Zeba Spin Desalting Columns (Thermo Fisher Scientific, #87773). Protein purity was assessed by SDS-PAGE followed by silver staining and Western blotting using anti-His monoclonal antibody. Protein concentrations were measured using the His-ELISA (Cayman Chemical; Cay10012445-96) with BSA as the standard.

### LRC-TriCEPS

To identify putative receptors or membrane-associated binding partners of ADAMTSL4-CT, we used LRC-TriCEPS (Dualsystems Biotech, cat. #P05201, study #R337) in collaboration with Dualsystems Biotech AG, following the manufacturer’s protocol.

Briefly, 300 μg of purified recombinant ADAMTSL4-CT-V5-6×His (ligand of interest), TRFE (positive control), or glycine (negative control) were dissolved in 150 μL of 25 mmol/L HEPES buffer (pH 8.2) and then conjugated to 1.5 μL of TriCEPS reagent and incubated at 20°C for 2 hours with constant agitation. Fully differentiated murine C2C12 myotubes were pretreated with 1.5 mmol/L sodium periodate (NaIO_4_) in PBS (pH 6.5) for mild oxidation of cell surface glycans, followed by incubation with TriCEPS-conjugated ligands for 2 hours at 4°C in the dark under gentle rotation. Cells were washed and collected in PBS, and pellets were shipped on dry ice to Dualsystems Biotech for downstream proteomic analysis.

Cells were lysed according to the standard LRC-TriCEPS protocol. Ligand–receptor complexes were isolated via solid-phase affinity purification. After extensive washing to remove nonspecific proteins, samples were reduced, alkylated, and digested with trypsin. Tryptic peptides were subjected to LC-MS/MS using a Q Exactive mass spectrometer (Thermo Fisher Scientific) equipped with an electrospray ion source. Peptides were separated on a 50-cm C18 column over a 90-minute gradient and acquired in data-dependent mode (TOP12).

Raw data were processed using Progenesis software for alignment and feature detection. Peptide spectra were identified using the Comet search engine and validated using the Trans-Proteomic Pipeline. Relative quantification was performed based on ion intensity. Differential protein abundance was determined using ANOVA, followed by multiple-testing correction. Significance was defined as log_2_ (FC) > 2 (FC >4) and −log_10_ (adjusted *P* value) > 2 (corresponding to adjusted *P* value < 0.01).

### IP Assay

To determine whether ADAMTSL4-CT selectively associates with LAP or mature TGFβ1, full-length proTGFβ1 was coexpressed with C-terminally V5- and 6×His-tagged ADAMTSL4-CT in HEK293T cells. In this construct, the N-terminal LAP and the C-terminal mature TGFβ1 moiety were selectively tagged with HA and FLAG epitopes, respectively (Supplementary Fig. S5A).

IPs were performed on whole-cell lysates using anti-HA or anti-FLAG antibodies to pull down either LAP or mature TGFβ1, followed by immunoblotting with anti-V5 or anti-His antibodies to detect co-precipitated ADAMTSL4-CT. Conversely, reciprocal IP using anti-V5 or anti-His antibodies to isolate ADAMTSL4-CT was followed by immunoblotting with anti-V5 or anti-His antibodies.

### NanoBiT Luciferase Complementation Assay

To assess proximity between ADAMTSL4-CT and the LAP, we used the *NanoBiT* luciferase complementation assay (Promega, cat. #N2014), which enables real-time detection of protein–protein proximity through reconstitution of a functional luciferase enzyme. The system consists of two subunits, SmBiT (small) and LgBiT (large), which generate luminescence when brought into close spatial proximity.

For interaction assays, LgBiT was fused to the C-terminus of LAP, and SmBiT was fused to the C-terminus of ADAMTSL4-CT (Fig. 5E). HEK293T cells were transiently transfected with the indicated constructs alone or in combination, and luminescence was measured as a readout of proximity between fusion partners.

To evaluate the impact of ADAMTSL4-CT on the latent LAP–TGFβ1 complex, a complementary NanoBiT configuration was used in which LgBiT was fused to LAP and SmBiT to the mature TGFβ1 domain within the full-length proTGFβ1 precursor (Fig. 5H). In this context, luminescence reflects proximity within the latent complex. Changes in luminescent signal upon ADAMTSL4-CT expression were interpreted as reflecting altered LAP–TGFβ1 proximity, consistent with modulation of latent complex stability.

### Western Blotting

Proteins were extracted from cells or tissues using ice-cold lysis buffer (50 mmol/L Tris-HCl, pH 7.2; 1 mmol/L EDTA; 1 mmol/L DTT; 150 mmol/L NaCl; 1% NP-40; 10 mmol/L NaF; 2 mmol/L Na_3_VO_4_; 1× cOmplete protease inhibitor cocktail, Roche; and 1× PhosSTOP phosphatase inhibitor cocktail, Roche), followed by centrifugation at 15,000 × *g* for 20 minutes at 4°C. Supernatants were collected and used for SDS–PAGE.

Proteins were resolved on 8% to 16% Tris-Glycine gradient SDS–PAGE gels (Bio-Rad, cat. #4561096) and transferred to polyvinylidene fluoride membranes (Bio-Rad, cat. #1620177). After blocking, membranes were incubated with the following primary antibodies: anti-ADAMTSL4 (1:500, Santa Cruz Biotechnology, sc-134654; RRID: AB_10609105), anti-ATGL (1:1,000, Cell Signaling Technology, #2138; RRID: AB_2167955), anti-VCP (1:1,000, Abcam, ab111740; RRID: AB_10861709), anti-β-actin (1:1,000, Sigma-Aldrich, A2228; RRID: AB_476697), anti-phospho-FoxO1 (Thr24)/FoxO3a (Thr32; 1:500, Cell Signaling Technology, #9464; RRID: AB_329842), anti-FoxO3a (1:500, Cell Signaling Technology, #2497; RRID: AB_836876), anti-phospho-STAT3 (Tyr705; 1:500, Cell Signaling Technology, #9145; RRID: AB_2491009), anti-STAT3 (1:500, Cell Signaling Technology, #12640; RRID: AB_2629499), anti-LC3B (1:1,000, Cell Signaling Technology, #3868; RRID: AB_2137707), anti-Bax (1:500, Santa Cruz Biotechnology, sc-20067; RRID: AB_626726), anti-FLAG (DYKDDDDK) Tag (1:1,000, Cell Signaling Technology, #14793; RRID: AB_2572291), anti-HA Tag (1:1,000, Cell Signaling Technology, #3724; RRID: AB_1549585), anti-perilipin-1 (1:1,000, Cell Signaling Technology, #3467; RRID: AB_2167270), anti-phospho-SMAD2 (Ser465/Ser467; 1:750, Cell Signaling Technology, #18338; RRID: AB_2798798), anti-SMAD2 (1:750, Cell Signaling Technology, #8685; RRID: AB_10889933), anti-vinculin (1:1,000, Cell Signaling Technology, #40211; RRID: AB_2728768), anti-6×His Tag (1:1,000, Abcam, ab5000; RRID: AB_304722), and anti-V5 Tag (1:2,000, Cell Signaling Technology, #13202; RRID: AB_2687461).

Secondary antibodies included mouse anti-rabbit IgG (1:2,000; light chain–specific, D4W3E, Cell Signaling Technology, #45262; RRID: AB_2799281) and rabbit anti-mouse IgG (1:2,000; light chain–specific, D3V2A, horseradish peroxidase–conjugated, Cell Signaling Technology, #58802; RRID: AB_2799549). Signal was visualized using enhanced chemiluminescence (Bio-Rad) on a ChemiDoc MP Imaging System (Bio-Rad, cat. #17001402).

### RNA Isolation and Quantitative PCR

Total RNA was isolated from cells or snap-frozen tissues using TRIzol Reagent (Thermo Fisher Scientific, #15596018) with mechanical disruption via TissueLyser II (Qiagen, #85300). Phase separation was performed using chloroform, followed by RNA precipitation with isopropanol and washes in 75% ethanol. RNA was resuspended in nuclease-free water and quantified.

RNA (1 μg) was treated with DNase and reverse-transcribed using the QuantiTect Reverse Transcription Kit (Qiagen, #205313) according to the manufacturer’s instructions. Quantitative PCR (qPCR) was performed using Power SYBR Green PCR Master Mix (Thermo Fisher Scientific, #4367659) or TaqMan Gene Expression Master Mix (Thermo Fisher Scientific, #4369514) on a QuantStudio 7 Flex Real-Time PCR System (Applied Biosystems, #4485695). Reactions were run in duplicate in 10 μL volumes for 40 cycles (95°C for 15 seconds, 60°C for 1 minute, and 72°C for 1 minute). Murine TaqMan probes: *Adamtsl4* (Mm00523242_m1), *Fbxo32* (Mm00499523_m1), *Trim63* (Mm01185221_m1), *Atg5* (Mm01187303_m1), *Becn1* (Mm01265461_m1), *Map1lc3* (Mm00782868_sH), *Gabarapl1* (Mm00457880_m1), *Bnip3l* (Mm00786306_s1), *Ctsl* (Mm00515597_m1), *Tgfb1* (Mm01178820_m1), *Ctgf* (Mm01192933_g1), *Tbp* (Mm01277042_m1), *Pmaip1* (Mm00451763_m1), *Ki67* (Mm01278617_m1), *Fabp4* (Mm00445878_m1), *Pdk4* (Mm01166879_m1), *Bbc3* (Mm00519268_m1), *Pnpla2* (Mm00503040_m1), *Lipe* (Mm00495359_m1), *Ccnd1* (Mm00432359_m1), *Pai1* (Mm00435858_m1), and *Plin5* (Mm00508852_m1). Murine primers, SYBR: *Cpt1*: F: gcctctctaagttacagccatttct, R: gctgctgctgggatctaacttc; *Fasn*: F: attggtggtgtggaccatggtc, R: cccagccttccatctcctg; *Ppargc1a*: F: gaagtggtgtagcgaccaatc, R: aatgagggcaatccgtctttca; *Col1a1*: F: cctcagggtattgctggacaac, R: cagaaggaccttgtttgccagg; *Col9a3*: F: caaaaaggcagcacaggagacc, R: agaccatcggaacctgcaatgc; *Spp1*: F: gcttggcttatggactgaggtc, R: ccttagactcaccgctcttcatg; *Tgfb1*: F: tgatacgcctgagtggctgtct, R: cacaagagcagtgagcgctgaa; *Tgfbr1*: F: tgctccaaaccacagagtaggc, R: cccagaacactaagcccattgc; *Tgfbr2*: F: cctactctgtctgtggatgacc, R: gacatccgtctgcttgaacgac. SYBR primers (human): *TGFB1*: F: tcgccagagtggttatctt, R: tagtgaacccgttgatgtcc; *CTGF/CCN2*: F: cttgcgaagctgacctggaaga, R: ccgtcggtacatactccacaga; *TGFBIP*: F: tgtgtgctgaagccatcgttg, R: ccggcttgtctgaaaaggtca; *TGM2*: F: ccatgaccagaacagcaacct, R: tgacctccgcaaagacaaag; *FBXO32*: F: gcagctgaacaacattcagatcac, R: cagcctctgcatgatgttcagt; *TRIM63*: F: cctgagaggcattgactttgg, R: cttcccttctgtggactcttcct; *FBXO21*: F: cctgagaggcattgactttgg, R: cttcccttctgtggactcttcct; *ACTINB*: F: gggaaatcgtgcgtgaca, R: ggactccatgcccagga; *RPL35A*: F: gggtacagcatcactcgga, and R: acgcccgagatgaaacag.

### RNA-seq Library Preparation and Sequencing

For RNA-seq of INTACT-sorted nuclei, 50 to 100 ng total RNA was processed using the VAHTS Stranded mRNA-seq Library Kit for Illumina (Vazyme) following the manufacturer’s protocol, omitting poly(A) selection. For whole-liver RNA-seq, 500 to 1,000 ng total RNA was processed using the NEBNext Ultra RNA Library Prep Kit for Illumina (NEB) according to the manufacturer’s instructions. Size selection was performed using AMPure XP beads (Beckman Coulter), and library quality was assessed on an Agilent Bioanalyzer 2100. Paired-end sequencing was conducted on an Illumina platform. The pathway analysis was done using the clusterProfiler package in R.

### Plasmids

The following constructs were used in this study: pLV[Exp]-Puro-EF1A>EGFP:T2A:mAdamtsl4 (VectorBuilder; expressing full-length mouse Adamtsl4) and pLV[Exp]-Puro-EF1A>mCherry:T2A:EGFP (VectorBuilder; control vector). For gene silencing, MISSION shRNA constructs (Sigma-Aldrich) targeting Adamtsl4 (TRCN0000080483, TRCN0000080484, and TRCN0000080485), Tgfb1/LAP (TRCN0000065997), and a nontargeting control (SHC016) were used.

The ADAMTSL4-CT-V5-6xHis expression plasmid (a gift from Dr. Clair Baldock, University of Manchester) encodes a C-terminally tagged, truncated form of ADAMTSL4 lacking the TSP1, cysteine-rich, and spacer domains. Constructs expressing HA-LAP-TGFβ1 and LAP-FLAG-TGFβ1 (gifts from Dr. Julian Nüchel and Dr. Markus Plomann) are based on the pcDNA4TO backbone and were modified to include epitope tags.

Plasmids encoding HA and FLAG tags were obtained from Addgene (#55182, RRID: Addgene_55182 and #55180, RRID: Addgene_55180, respectively).

Synthetic reporter constructs, including pLVX-Puro-Pro-TGFβ1-Nanoluc (GBS230125CSB), pLVX-Puro-Pro-TGFβ1-LAPtagged-LgBit, and pLVX-Puro-BM40-ADAMTSL4CT-GS-SmBit-myc-HIS (GBS230125DSB), were generated in collaboration with BioCat GmbH.

### Figure Preparation

Graphs were generated using GraphPad Prism (version 10, GraphPad Software; RRID: SCR_002798). Figure panels were assembled in Microsoft PowerPoint (Microsoft Corporation). Schematic illustrations were created using BioRender (BioRender.com; RRID: SCR_018361).

### Quantification and Statistical Analysis

All statistical analyses were performed using GraphPad Prism (version 10, GraphPad Software; RRID: SCR_002798). Data distribution was assessed by visual inspection together with Shapiro–Wilk and Kolmogorov–Smirnov normality tests. Statistical significance was determined using the following tests, as appropriate: two-tailed unpaired *t* test, multiple unpaired *t* tests with Welch correction, one-way ANOVA followed by Tukey multiple-comparisons test, two-way ANOVA or mixed-effects model (REML) followed by Tukey or Holm–Šídák multiple-comparisons test, and nonparametric two-tailed Mann–Whitney tests. Details of the statistical tests used for each experiment are provided in the corresponding figure legends. For *in vitro* and preclinical experiments, data are presented as the mean ± SEM unless otherwise indicated.

For human circulating biomarker analyses, datasets were systematically evaluated for distribution and potential outliers. Normality was assessed by visual inspection together with Shapiro–Wilk testing. ELISA-based biomarker measurements (e.g., ADAMTSL4, GDF15, and TGFβ1) showed right-skewed distributions and were therefore analyzed primarily using two-tailed Mann–Whitney tests. As sensitivity analyses, ELISA values were additionally log_10_-transformed and analyzed using Welch *t* test. ROUT outlier detection (Q = 1%) was applied only as a sensitivity analysis and did not affect the primary analyses. Human biomarker data are displayed as Tukey box-and-whisker plots with individual data points overlaid, with center lines indicating the median and boxes indicating the interquartile range. Correlation analyses involving non-Gaussian or ordinal variables (e.g., cachexia grade) were performed using two-tailed Spearman rank correlation. ROC curve analyses were performed to assess biomarker performance in distinguishing cachectic versus noncachectic patients, and area under the curve values were calculated. For survival analyses in the TRACERx cohort, patients were stratified based on the cohort median of circulating ADAMTSL4 levels (NPX), and Kaplan–Meier curves were compared using the log-rank (Mantel–Cox) test. No data points were excluded unless predefined experimental criteria were met (e.g., humane endpoints in animal studies). Statistical significance was defined as *P* < 0.05.

## Supplementary Material

Supplementary Tables S1-S4 Table S1: Characterization of colorectal cancer patients. Table S2: Characterization of Lung cancer adenocarcinoma stratified from NSCLC cohort, cancer cachexia cohort 3 (29). Table S3: Enrichment of proteins obtained in Ligand-receptor capture followed LC-MS/MS analysis. Table S4: RRID Addendum.

Supplementary Figures S1-S6 Supplementary Fig. S1. ADAMTSL4 is enriched in conditioned media of cachexia-inducing cancer cell lines and is associated with systemic metabolic alterations in patients with cancer. Supplementary Fig. S2. Overexpression of ADAMTSL4 converts non-cachexia-inducing cells into cachexia-inducing cells. Supplementary Fig. S3. Stable knockdown of ADAMTSL4 in cachexia-inducing cells attenuates their muscle-wasting capacity in vitro. Supplementary Fig. S4. Stable knockdown of ADAMTSL4 in cachexia-inducing tumor cells attenuates in vitro and in vivo adipocyte wasting and prolongs cachexia-related survival. Supplementary Fig. S5. ADAMTSL4 targets LAP of latent TGF-β1 complex to promote myotube atrophy via TGF-β latency activation and TGFβR-dependent signaling. Supplementary Fig. S6. ADAMTSL4 induces wasting of murine and human white adipocytes via local activation of TGF-β1–TGFBR1 signaling, without altering circulating TGF-β1 levels.

Supplementary Data S1-S9 Excel spreadsheets with complete GO-enrichment analyses and TRICEPS-MS data

## Data Availability

Sequencing data have been deposited in the Gene Expression Omnibus under accession number GSE304945. Any additional information required to reanalyze the data reported in this article is available from the lead contact upon reasonable request. A comprehensive list of reagents and resources, including RRIDs, is provided in the Key Resources Table (Supplementary Table S4).
