# Supplementary material for: Tumor-Secreted ADAMTSL4 Activates Latent TGFβ1 to Drive Cancer Cachexia
Source: Cancer Discov. 2026 Sep 1;16(10):2172–96. doi: 10.1158/2159-8290.CD-26-0045 (PMC13628105; doi:10.1158/2159-8290.CD-26-0045)
Supplement: Supplementary file 1 [file cd-26-0045_supplementary_figures_s1-s6_suppsf1.pdf]

## Supplementary Figure S1.

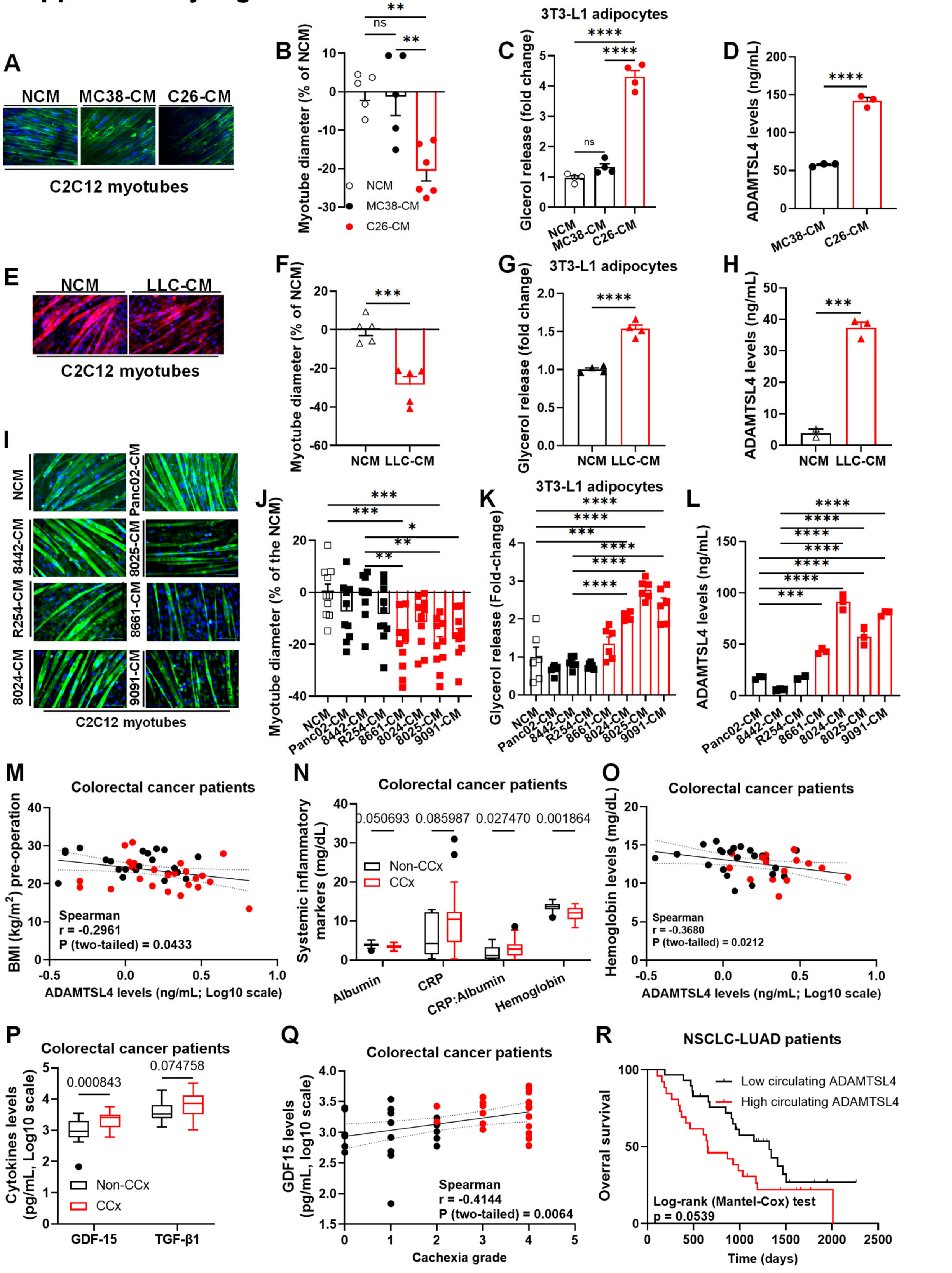


**Supplementary Fig. S1. ADAMTSL4 is enriched in conditioned media of cachexia-inducing cancer cell lines and is associated with systemic metabolic alterations in patients with cancer.** (**A–D**) Conditioned media (CM) from tumor cell lines were assessed for cachexia-inducing properties in *vitro*. Representative images (**A**) and quantification of C2C12 myotube diameter (**B**), glycerol release from 3T3-L1 adipocytes (**C**), and ADAMTSL4 protein levels measured by ELISA (**D**) in non-conditioned medium (NCM), CM from non-cachexia-inducing MC38 cells (MC38-CM), or CM from cachexia-inducing C26 cells

(C26-CM). (**E–H**) Representative images (**E**) and quantification of C2C12 myotube diameter (**F**), glycerol release from 3T3-L1 adipocytes (**G**), and ADAMTSL4 protein levels (**H**) in NCM or CM from cachexia-inducing LLC cells (LLC-CM). (**I–L**) Representative images (**I**) and quantification of C2C12 myotube diameter (**J**), glycerol release from 3T3-L1 adipocytes (**K**), and ADAMTSL4 protein levels (**L**) in CM from murine pancreatic ductal adenocarcinoma (PDAC) cell lines compared with NCM. (**M**) Spearman correlation between circulating ADAMTSL4 levels (ELISA, log10 scale) and body mass index (BMI) in colorectal cancer (CRC) patients (n = 47 XY pairs; r = −0.2961, P = 0.0433). (**N**) Circulating systemic biomarkers in CRC patients, including albumin, C-reactive protein (CRP), CRP/albumin ratio, and hemoglobin, shown for non-cachectic (Non-CCx) and cachectic (CCx) patients. (**O**) Spearman correlation between circulating ADAMTSL4 levels (ELISA, log10 scale) and hemoglobin levels in CRC patients (n = 39 XY pairs; r = −0.3680, P = 0.0212). (**P**) Circulating cytokine levels (GDF-15 and TGF-β1; pg/mL, log10 scale) in CRC patients stratified by cachexia status (Non-CCx versus CCx). Mean ± SEM: GDF-15, Non-CCx = 1298 ± 182 pg/mL, CCx = 2364 ± 271 pg/mL; TGF-β1, Non-CCx = 5668 ± 1230 pg/mL, CCx = 10703 ± 2106 pg/mL. (**Q**) Spearman correlation between circulating GDF-15 levels (log10 scale) and cachexia grade in CRC patients (n = 42 XY pairs; r = 0.4144, P = 0.0064). (**R**) Kaplan–Meier analysis of overall survival in lung adenocarcinoma (LUAD) patients from the TRACERx cohort stratified by high versus low circulating ADAMTSL4 (median NPX as cutoff). Data are presented as mean ± SEM for *in vitro* experiments (**B–D, F–H, and J–L**). For human biomarker comparisons (**N and P**), data are shown as box-and-whisker plots with individual data points overlaid. Boxes indicate the interquartile range, center lines indicate the median, and whiskers indicate Tukey whiskers (1.5 × IQR). ELISA-based measurements in CRC patients (**M, O, and P**) are shown on a log10-transformed scale where indicated. **Statistical analysis**: one-way ANOVA with Tukey's multiple-comparisons test (**B–D and J–L**); two-tailed unpaired t test (**F–H**). Human data were systematically evaluated for normality and outliers (ROUT, Q = 1%). For ELISA-based comparisons, distributions were right-skewed; therefore, two-tailed Mann–Whitney tests were used as the primary analysis, with Welch's t test on log10-transformed data performed as sensitivity analysis (see Methods). Group comparisons of circulating biomarkers (**N and P**) were analyzed using two-tailed Mann–Whitney tests. Correlation analyses (**M, O, and Q**) were performed using two-tailed Spearman rank correlation. Kaplan–Meier survival curves (**R**) were compared using the log-rank (Mantel–Cox) test. Significance is denoted as *P < 0.05, **P < 0.01, ***P < 0.001, ****P < 0.0001; n.s., not significant.

## Supplementary Figure S2.

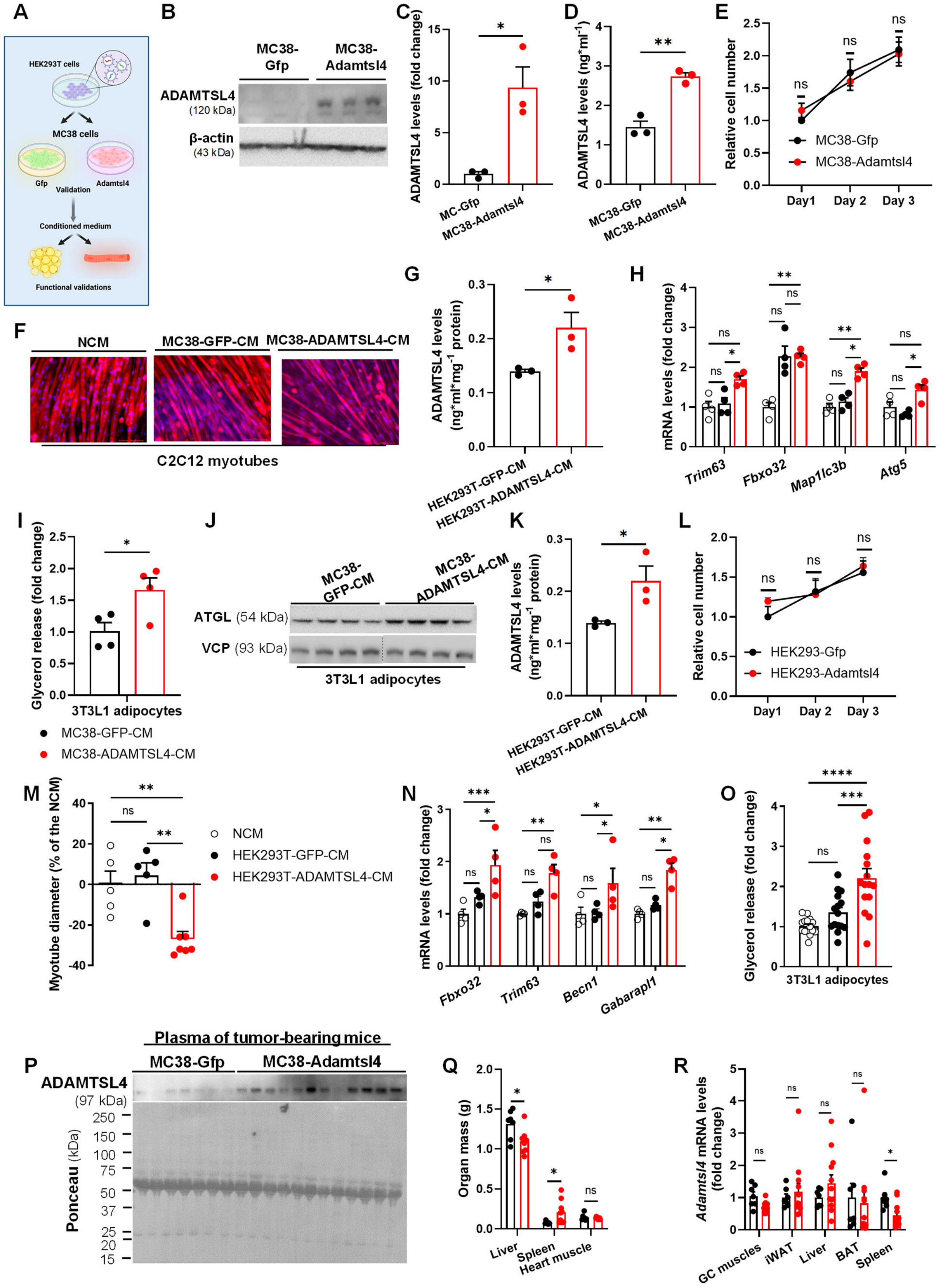


**Supplementary Fig. S2. Overexpression of ADAMTSL4 converts non-cachexia-inducing cells into cachexia-inducing cells.** (**A**) Schematic overview of lentiviral production, generation of MC38 cell lines stably expressing Adamtsl4 or Gfp, and the experimental pipeline for conditioned medium (CM) production and functional assays in adipocytes and myotubes. The diagram was created using BioRender. Machado,

J. (2026) https://BioRender.com/q1z1l3n. (**B and C**) ADAMTSL4 protein levels in whole-cell lysates (WCL) of MC38-Gfp and MC38-Adamtsl4 cells assessed by western blot. (**D**) ADAMTSL4 protein levels in CM determined by ELISA. (**E**) Growth curve of MC38-Adamtsl4 (red) and MC38-Gfp (black) cells. (**F**) Representative images, (**G**) quantification of myotube diameter, and (**H**) relative mRNA expression of proteolytic markers (*Trim63, Fbxo32, Map1lc3, Atg5*) in C2C12 myotubes treated with non-conditioned medium (NCM), MC38-Gfp-CM, or MC38-Adamtsl4-CM. (**I**) Glycerol levels in the medium of 3T3-L1 adipocytes and (**J**) ATGL protein levels by western blot in 3T3-L1 adipocytes treated with CM from MC38-Gfp or MC38-Adamtsl4 cells. (**K**) ADAMTSL4 protein levels in CM and (**L**) growth curve of stably Adamtsl4-overexpressing HEK293T cells (HEK293T-Adamtsl4) compared with HEK293T-Gfp cells. (**M**) Quantification of myotube diameter and (**N**) relative mRNA expression of proteolytic genes (*Fbxo32, Trim63, Becn1, Gabarapl1*) in C2C12 myotubes treated with NCM, HEK293T-Gfp-CM, or HEK293T-Adamtsl4-CM. (**O**) Glycerol release into the medium from 3T3-L1 adipocytes after 24 h treatment with HEK293T-derived CM. (**P–R**) *In vivo* validation using MC38-Gfp (n = 7) or MC38-Adamtsl4 (n = 9–12) tumor-bearing mice. (**P**) Plasma ADAMTSL4 protein levels assessed by western blot. (**Q**) Relative Adamtsl4 mRNA levels in tumor, liver, gastrocnemius (GC) muscle, and inguinal white adipose tissue (iWAT) assessed by qPCR. (**R**) Organ mass measurements in tumor-bearing mice. Data are presented as mean ± SEM. Each dot represents one biological replicate. **Statistical analysis**: two-way mixed-effects ANOVA (REML) with Sidak's multiple-comparisons test (**E, L**); two-tailed unpaired t test (**C, D, I, K**); one-way ANOVA with Tukey's multiple-comparisons test (**G, H, M, N, O, Q, R**). Significance is denoted as $^*P < 0.05$, $^{**}P < 0.01$, $^{***}P < 0.001$, $^{****}P < 0.0001$; n.s., not significant.

## Supplementary Figure S3.

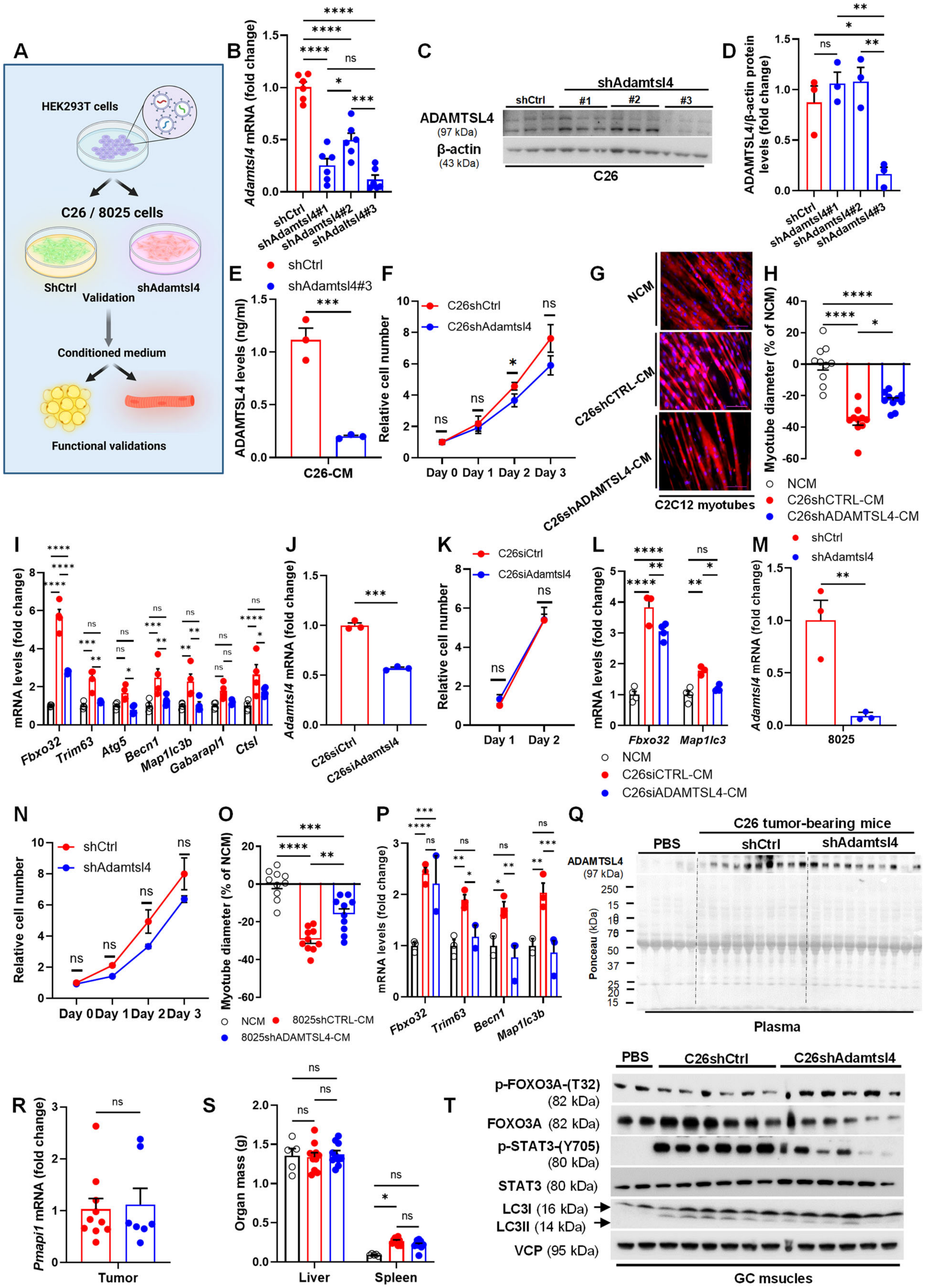

**Supplementary Fig. S3. Stable knockdown of ADAMTSL4 in cachexia-inducing cells attenuates their muscle-wasting capacity in vitro.** (**A**) Schematic overview of lentiviral production, generation of C26 cell lines with stable Adamtsl4 knockdown (C26shAdamtsl4) or control (C26shCtrl), and the pipeline for conditioned medium (CM) production and in vitro functional assays using C2C12 myotubes and 3T3-L1

adipocytes. The diagram was created using BioRender. Machado, J. (2026) https://BioRender.com/kdmq7r7. (**B**) Relative Adamtsl4 mRNA levels in C26shCtrl and C26shAdamtsl4 cells determined by qPCR. (**C, D**) ADAMTSL4 protein levels in whole cell lysates (WCL) assessed by western blot analysis. (**E**) ADAMTSL4 protein levels in CM measured by ELISA. (**F**) Growth curve of C26shCtrl and C26shAdamtsl4 cells. (**G**) Representative images, (**H**) quantification of myotube diameter, and (**I**) relative mRNA levels of key proteolytic genes in C2C12 myotubes treated with NCM, C26shCtrl-CM, or C26shAdamtsl4-CM, including *Trim63, Fbxo32* (proteasome pathway), and *Atg5, Becn1, Map1lc3, Gabarapl1, and Ctsl* (autophagy pathway). (**J**) Relative *Adamtsl4* mRNA levels and (**K**) growth curve of C26 cells transiently transfected with siRNA targeting *Adamtsl4* (C26siAdamtsl4) or control siRNA (C26siCtrl). (**L**) Relative *Fbxo32 and Map1lc3* mRNA levels in C2C12 myotubes treated with NCM, C26siCtrl-CM, or C26siAdamtsl4-CM. (**M**) Relative Adamtsl4 mRNA levels and (N) growth curve of 8025 cells with stable Adamtsl4 knockdown (8025shAdamtsl4) or control (8025shCtrl). (**O**) Quantification of myotube diameter, and (**P**) relative expression of *Trim63, Fbxo32, Becn1, and Map1lc3* in C2C12 myotubes treated with NCM, 8025shCtrl-CM, or 8025shAdamtsl4-CM. (**Q–T**) In vivo validation using PBS-injected mice (n = 5), or mice bearing C26shCtrl (n = 10) or C26shAdamtsl4 (n = 9) tumors. (**Q**) Western blot analysis of ADAMTSL4 protein levels in the plasma of tumor-bearing mice. (**R**) Relative *Pmap1* mRNA levels in tumors measured by qPCR. (**S**) Organ mass measurements. (**T**) Western blot analysis of p-FOXO3A and p-STAT3 along with their respective total protein levels, and LC3 protein levels, in gastrocnemius (GC) muscle from tumor-bearing mice. Data are presented as mean ± s.e.m. Each dot represents one biological replicate. Statistical analysis: (B, D, H, I, L, O, P, S) one-way ANOVA with Tukey's multiple-comparisons test; (F, K, N) two-way mixed-effects ANOVA (REML) with Šídák's multiple comparisons test; (E, J, M, R) two-tailed unpaired t-test. *P < 0.05; **P < 0.01; ***P < 0.001; ****P < 0.0001; n.s., not significant.

## Supplementary Figure S4.

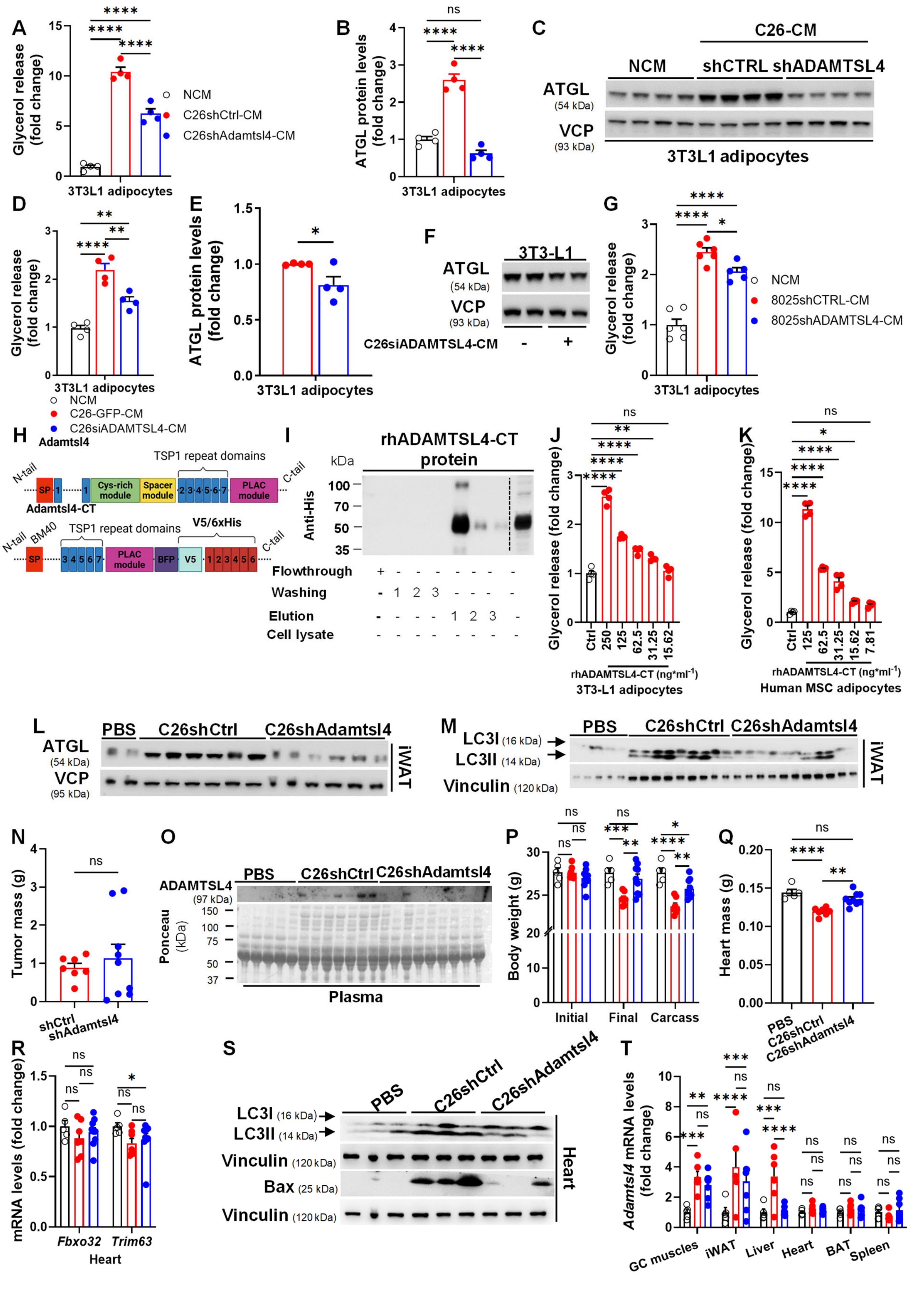

**Supplementary Fig. S4. Stable knockdown of ADAMTSL4 in cachexia-inducing tumor cells attenuates in vitro and in vivo adipocyte wasting and prolongs cachexia-related survival.** (**A**) Glycerol release into the medium of 3T3-L1 adipocytes, (**B**) quantitative analysis, and (**C**) ATGL protein levels in whole cell lysates (WCL) of 3T3-L1 adipocytes treated for 24 h with NCM, C26shCtrl-CM, or

C26shAdamtsl4-CM. (**D**) Glycerol release, (**E**) quantification, and (**F**) ATGL protein levels in 3T3-L1 adipocytes treated for 24 h with CM from transiently transfected C26siCtrl or C26siAdamtsl4 cells. (**G**) Glycerol levels in the medium of 3T3-L1 adipocytes treated for 24 h with NCM, 8025shCtrl-CM, or 8025shAdamtsl4-CM. (**H**) Schematic representation of the domain architecture of full-length (FL) human ADAMTSL4 and its truncated recombinant form (ADAMTSL4-CT). The diagram was created using BioRender. Machado, J. (2026) https://BioRender.com/4id59bx. (**I**) Western blot validation of His-tagged recombinant human ADAMTSL4-CT (rhADAMTSL4-CT) protein in Expi293F cell-conditioned medium and corresponding cell lysate. (**J**) Glycerol release into the medium of 3T3-L1 adipocytes treated with increasing concentrations of rhADAMTSL4-CT for 24 h. (**K**) Glycerol release from human MSC-derived adipocytes treated with increasing concentrations of rhADAMTSL4-CT for 24 h. (**L–M**) *In vivo* validation in mice inoculated subcutaneously with PBS, C26shCtrl, or C26shAdamtsl4 cells. (**L**) ATGL and (**M**) LC3 protein levels in iWAT tissue lysates determined by western blot analysis. (**N–T**) Independent *in vivo* cohort to evaluate the impact of tumor-derived ADAMTSL4 on cachexia-related survival. (**N**) Tumor mass, (**O**) plasma ADAMTSL4 protein levels by western blot, (**P**) initial and final body weight (BW), and carcass weight. (**Q**) Heart mass and (**R**) relative mRNA expression of Trim63 and Fbxo32 in the heart by qPCR. (**S**) Representative western blot images of LC3-**I**, LC3-**II** (autophagy markers), and Bax (apoptosis marker) in heart tissue from PBS-, C26shCtrl-, or C26shAdamtsl4-injected mice. (**T**) *Adamtsl4* mRNA expression across tissues (tumor, liver, muscle, iWAT) in PBS, C26shCtrl, and C26shAdamtsl4 mice. Data are presented as mean ± s.e.m. Each dot represents one biological replicate. Statistical analysis: (**A–C, G, J, K, Q, T**) one-way ANOVA with Tukey's multiple-comparisons test; (**E, N**) two-tailed unpaired t-test; (**P, R**) two-way ANOVA or mixed-effects model (REML) with Tukey's multiple-comparisons test. *P < 0.05; **P < 0.01; ***P < 0.001; ****P < 0.0001; n.s., not significant.

## Supplementary Figure S5.

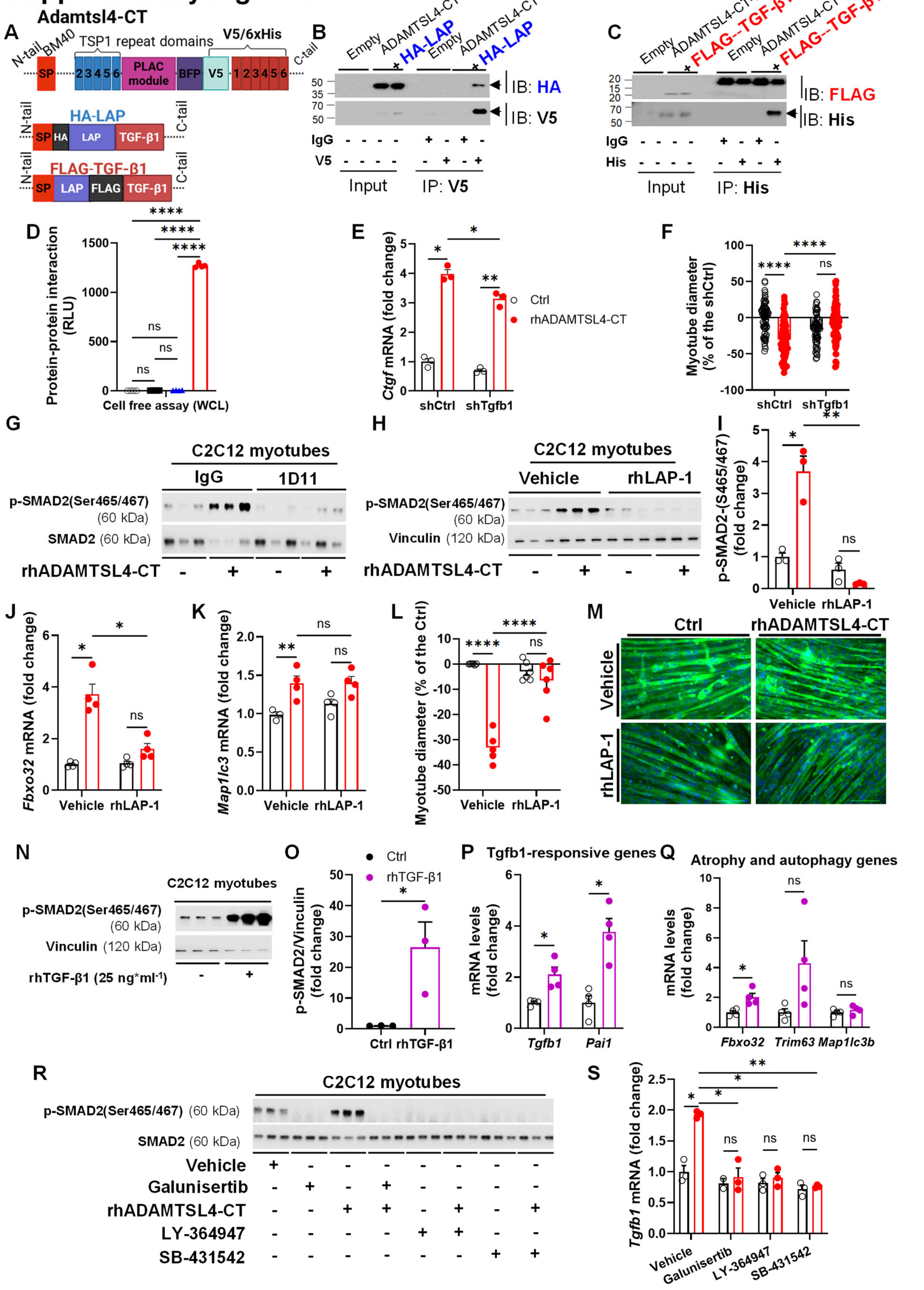


**Supplementary Fig. S5. ADAMTSL4 targets LAP of latent TGF-β1 complex to promote myotube atrophy via TGF-β latency activation and TGFβR-dependent signaling.** (**A**) Schematic representation of the expression constructs used for interaction studies. ADAMTSL4-CT was expressed as a tagged construct carrying V5 and 6×His epitopes. Full-length proTGF-β1 was engineered with epitope tags that

distinguish the latency-associated peptide (LAP; HA-tagged) from mature TGF-β1 (FLAG-tagged), enabling selective interrogation of the two moieties in co-immunoprecipitation assays. The diagram was created using BioRender. Machado, J. (2026) https://BioRender.com/gisnh9z. (**B–C**) Co-immunoprecipitation assays performed in HEK293T cells transiently expressing the tagged constructs, followed by immunoprecipitation from whole-cell lysates (WCL). (**B**) Immunoprecipitation of HA-LAP specifically co-precipitated ADAMTSL4-CT, demonstrating interaction with LAP. (**C**) In contrast, immunoprecipitation of FLAG-tagged mature TGF-β1 did not detect interaction with ADAMTSL4-CT despite clear input controls, supporting selective binding of ADAMTSL4 to LAP rather than mature TGF-β1. (**D**) NanoBiT protein–protein interaction assay performed in a cell-free system using whole-cell lysates (WCL), complementary to the live-cell NanoBiT interaction assay shown in the main **Figure 5**, further confirming interaction between ADAMTSL4 and LAP. (**E–F**) Complementary functional validation of proTGF-β1 dependency in C2C12 myotubes. Lentiviral shRNA-mediated knockdown of proTgfb1 attenuated ADAMTSL4-induced responses, as shown by reduced *Ctgf* mRNA expression (**E**) and prevention of myotube atrophy, quantified by myotube diameter (**F**), complementing the corresponding results presented in the main **Figure 5**. (**G**) Representative Western blot image of phosphorylated SMAD2 (p-SMAD2) in C2C12 myotubes treated with vehicle or recombinant human ADAMTSL4-CT (rhADAMTSL4-CT; 250 ng/mL) in the presence or absence of the TGF-β-neutralizing antibody 1D11 (1.5 µg/mL), complementing the quantified results shown in the main **Figure 5**. (**H–M**) Functional blockade of LAP engagement in C2C12 myotubes treated with rhADAMTSL4-CT in the presence or absence of recombinant human LAP (rhLAP; 500 $ng/mL^{-1}$). rhLAP suppressed ADAMTSL4-induced TGF-β pathway activation and downstream catabolic effects, as assessed by SMAD2 phosphorylation, TGF-β-responsive and atrophy/autophagy-related gene expression, myotube diameter, and representative immunofluorescence images. Together, these data further support that LAP engagement is required for ADAMTSL4 activity. (**N–Q**) Positive-control experiments in which recombinant human TGF-β1 (25 $ng/ml^{-1}$) was applied to C2C12 myotubes to test whether exogenous TGF-β1 recapitulates the effects induced by ADAMTSL4. Recombinant TGF-β1 increased SMAD2 phosphorylation (**N-O**) and induced canonical TGF-β target genes (**P**) as well as atrophy/autophagy-related genes (**Q**), supporting the conclusion that TGF-β1 signaling is sufficient to reproduce the downstream program triggered by ADAMTSL4. (**R-S**) Receptor-level validation of pathway dependency. Pharmacological inhibition of TGFBR1 using galunisertib (10 µM), LY-364947 (10 µM), or SB-431542 (10 µM) suppressed ADAMTSL4-induced signaling responses, as shown by representative Western blot analysis (**R**) and Tgfb1 mRNA expression (**S**), further supporting TGFBR1 dependence of ADAMTSL4-mediated effects.
Data are presented as mean ± SEM. Each dot represents one biological replicate. **Statistical analysis**: one-way ANOVA followed by Tukey's multiple-comparisons test (**D**); two-way repeated-measures ANOVA (matching both factors, without assuming sphericity) followed by Tukey's multiple-comparisons test (**E–F, H–M, S**); two-tailed unpaired t test (**O**); and multiple two-tailed unpaired t tests with correction for multiple comparisons (**P–Q**). Significance is denoted as *P < 0.05, **P < 0.01, ***P < 0.001, ****P < 0.0001; n.s., not significant.

## Supplementary Figure S6.

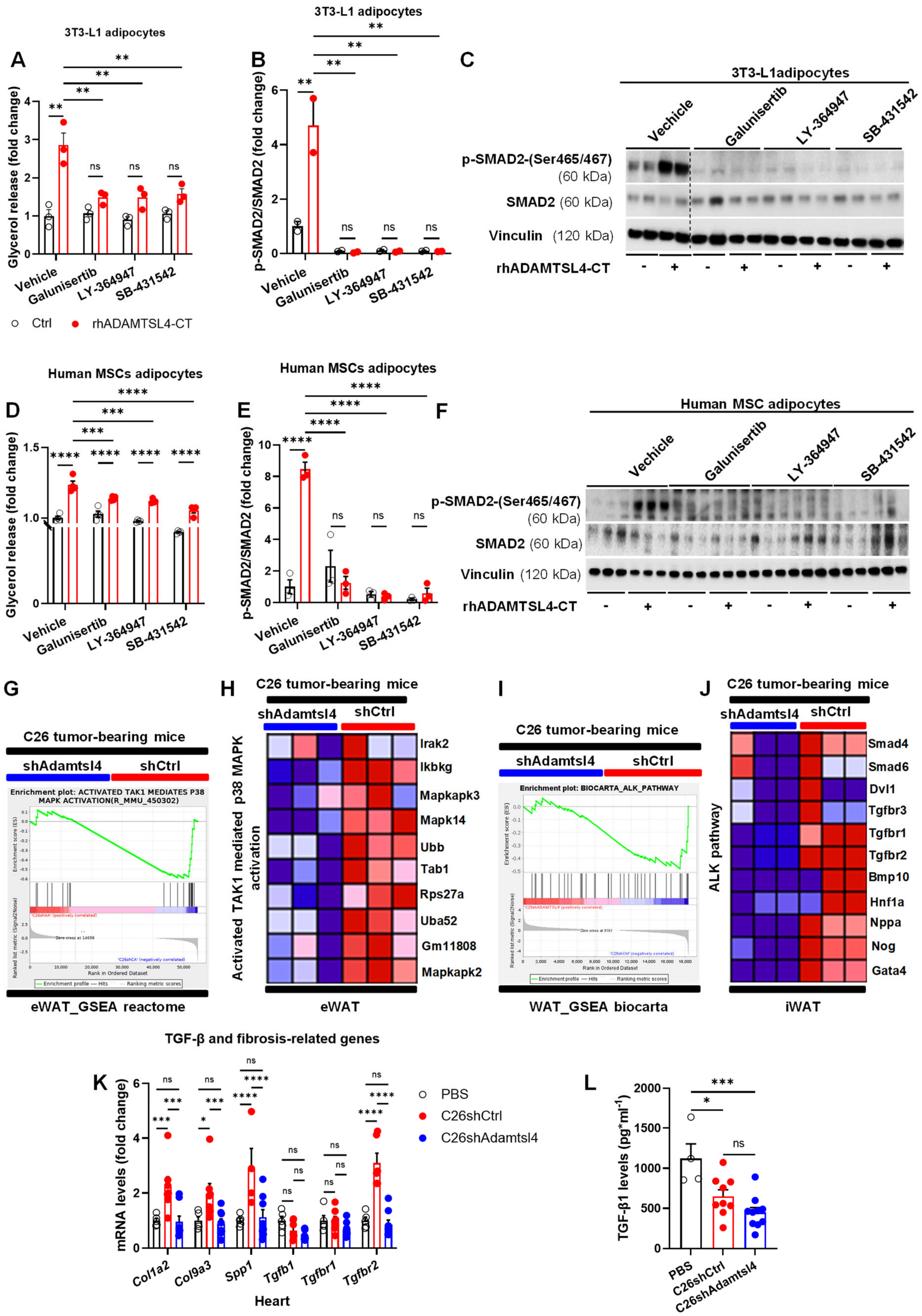


**Supplementary Fig. S6. ADAMTSL4 induces wasting of murine and human white adipocytes via local activation of TGF-β1–TGFBR1 signaling, without altering circulating TGF-β1 levels.** (**A**) Glycerol release into the media of murine 3T3-L1 adipocytes, (**B**) quantification of lipolysis, and (**C**) representative Western blot of SMAD2 phosphorylation in whole-cell lysates (WCL) of 3T3-L1 adipocytes treated for 24 h

with vehicle or rhADAMTSL4-CT (recombinant human ADAMTSL4 C-terminal domain; 250 ng·mL$^{-1}$), in the presence or absence of TGFBR1 inhibitors: galunisertib (10 µM), LY-364947 (10 µM), or SB-431542 (10 µM). (**D**) Glycerol release, (**E**) quantification of lipolysis, and (**F**) representative SMAD2 phosphorylation in human MSC-derived adipocytes treated for 24 h with vehicle or rhADAMTSL4-CT in the presence or absence of the indicated TGFBR1 inhibitors. (**G**) Gene Set Enrichment Analysis (GSEA) enrichment plot for the noncanonical TGF-β signaling pathway “Activated TAK1 mediates p38 MAPK activation”, and (**H**) corresponding heatmap of the top 10 differentially expressed genes (DEGs) in epididymal white adipose tissue (eWAT) from C26shCtrl versus C26shAdamtsl4 tumor-bearing mice. (**I**) GSEA enrichment plot for the canonical TGF-β pathway “ALK signaling in TGF-β receptor pathway”, and (**J**) corresponding heatmap of the top 10 DEGs in inguinal white adipose tissue (iWAT) from the same mouse cohorts. (**K**) Relative expression of fibrosis-related genes (Col1a2, Col9a3, Spp1, Tgfb1, Tgfb2, Tgfbr2) in the hearts of PBS-injected, C26shCtrl, and C26shAdamtsl4 tumor-bearing mice determined by qPCR. (**L**) Circulating TGF-β1 levels measured by ELISA in plasma from PBS-injected mice and tumor-bearing mice with or without stable Adamtsl4 knockdown in C26 cells. Data are shown as mean ± SEM unless indicated otherwise. Each dot represents one biological replicate. Statistical analysis: (**A–B, D–E**) two-way mixed-effects model with Tukey’s or Šídák’s multiple-comparisons test; (**C, F**) representative blots from n = 3 biological replicates; (**K**) two-way ANOVA or mixed-effects model (REML) with Tukey’s multiple-comparisons test; (**L**) one-way ANOVA with Tukey’s multiple-comparisons test. Significance is denoted as ****$P < 0.0001$; ***$P < 0.001$; **$P < 0.01$; *$P < 0.05$; n.s., not significant.
