# Supplementary material for: Tumor-Secreted ADAMTSL4 Activates Latent TGFβ1 to Drive Cancer Cachexia
Source: Cancer Discov. 2026 Sep 1;16(10):2172–96. doi: 10.1158/2159-8290.CD-26-0045 (PMC13628105; doi:10.1158/2159-8290.CD-26-0045)
Supplement: Supplementary file 2 [file cd-26-0045_supplementary_tables_s1-s4_suppst1.pdf]

Table S1: Characterization of colorectal cancer patients.

	Non-CCx	CCx	p-value
**Clinical information**			
Male (n)	11	15	
Female (n)	9	12	
Age	58.65 ± 2.166	63.37 ± 2.967	0.0901
Relative BW changes (%)	- 0.6314 ± 0.4486	- 12.44 ± 1.358	**< 0.0001 (****)**
BMI (kg/m2)	25.23 ± 0.6302	22.41 ± 0.7810	0.0066 (**)
BMI changes (kg/m2)	0.1585 ± 0.1226	- 3.172 ± 0.3410	**< 0.0001 (****)**
**Tumour site**			
Colorectal	20	27	
**UICC Tumour stage (n)**			
I	4	4	
II	5	8	
III	4	6	
IV	4	5	
Non-classified	3	4	

Characterization of the non-cancer cachexia (non-CCx) and cancer cachexia (CCx) patients. UICC, Union for International Cancer Control. Relative body weight changes (BW; %), body mass index (BMI; kg/m^2), and BMI changes (kg/m^2), were measured in the day of the surgery or blood sample relatively to the previous 6 months. Cachexia was defined according to Fearon, K. et al (29). Data are mean ± SEM. Data are mean ± SEM. Statistical analyses were performed using an unpaired two-tailed Mann-Whitney U.

Table S2: Characterization of Lung cancer adenocarcinoma stratified from NSCLC cohort, cancer cachexia cohort 3 (29).

	Non-CCx	CCx	p-value
**Clinical information**			
Male (n)	14	8	
Female (n)	16	6	
Age	66.00 ± 1.815	69.43 ± 3.607	0.4059
Relative BW change (%)	1.838 ± 0.9754	-10.17 ± 1.237****	**<0.0001**
BMI (kg/m2)	27.29 ± 0.7147	25.69 ± 1.52	0.3528
BMI change (kg/m2)	0.4613 ± 0.2469	-2.932 ± 0.3945****	**<0.0001**
**Tumour site**			
LUAD	30	14	
LUSC	9	8	
Other	10	1	
**UICC Tumour stage - LUAD**			
I	9	4	
II	7	1	
III	14	9	
IV	-	-	
Non-classified	-	-	
**Pearson correlation analysis**			
ADAMTSL4 vs BMI change	Number of XY Pairs = 44	r = 0.4133	**0.0053**

Characterization of the non-cancer cachexia (non-CCx) and cancer cachexia (CCx) patients. Data are mean ± SEM. Statistical analyses were performed using an unpaired two-tailed t test with Welch's correction analysis and two-tailed Spearman correlation analysis.

Table S3: Enrichment of proteins obtained in Ligand-receptor capture followed LC-MS/MS analysis.

			ADAMTSL4 vs transferrin		ADAMTSL4 vs glycine	
	Accession	Protein description	**Log2(FC)**	**adj pValue**	**Log2(FC)**	**adj pValue**
TGFB1	P04202	Transforming growth factor beta-1 proprote	-6.32	2.25E-05	-6.32	2.71E-04
I17RC	Q8K4C2	Interleukin-17 receptor C	-2.63	1.15E-02	0.51	1.74E-02
TPA	P11214	Tissue-type plasminogen activator	-2.48	1.43E-04	-1.34	1.31E-03
MIRO1	Q8BG51	Mitochondrial Rho GTPase 1	-2.20	3.07E-04	0.91	2.75E-03
BTD	Q8CIF4	Biotinidase	-1.01	4.28E-02	-1.90	1.71E-03
RAP1A*	P62835	Ras-related protein Rap-1A	-3.72	1.31E-01	-0.67	5.01E-02
ABCBB*	Q9QY30	Bile salt export pump	-3.51	1.13E-01	-0.41	5.16E-01
SCMC1*	Q8BMD8	Calcium-binding mitochondrial carrier proteir	-2.81	8.77E-02	-0.68	4.04E-01
WISP1*	O54775	WNT1-inducible-signaling pathway protein 1	-2.65	1.66E-01	-0.41	2.06E-02
DTBP1*	Q91WZ8	Dysbindin	-2.58	1.31E-01	-0.51	3.99E-03
SNG2*	O55101	Synaptogyrin-2	-2.17	1.97E-01	-1.59	2.58E-01
KAPCA*	P05132	cAMP-dependent protein kinase catalytic su	-2.02	1.06E-01	-0.21	5.90E-01
DPEP1*	P31428	Dipeptidase 1	-1.98	1.04E-01	0.66	2.86E-02
ROCK2*	P70336	Rho-associated protein kinase 2	-0.31	6.54E-01	-3.45	5.51E-02
GKN3*	Q9D0T7	Gastrokine-3	1.05	8.48E-01	-2.00	1.34E-02

* Proteins found to be enriched but missed the p-value. Protein TGFB1, identified with just one peptide was significantly enriched in both quantitative comparisons. TPA and SNG2, identified with just one were found to be enriched in the ligand samples compared to the transferrin samples and borderline enriched compared to the glycine samples**.**

Table S4: RRID Addendum

Machado J., et al. Tumor-secreted ADAMTSL4 activates latent TGF-β1 to drive cancer cachexia

KEY RESOURCES TABLE

REAGENT or RESOURCE	SOURCE	IDENTIFIER
**Experimental models: Cell lines**		
C2C12 (mouse myoblast)	ATCC	Cat#: CRL-1772; RRID:CVCL_0188
3T3-L1 (mouse preadipocyte)	ATCC	Cat#: CL-173; RRID:CVCL_0123
HEK293T	ATCC	Cat#: CRL-3216; RRID:CVCL_0063
Human primary skeletal muscle cells	ATCC	Cat# PCS-950-010; RRID: N/A
MC38 (mouse colon adenocarcinoma)	Merck-Millipore	Cat#: SCC172; RRID:CVCL_B288
C26 (mouse colon carcinoma)	German Cancer Research Center (Deutsches Krebsforschungszentrum, DKFZ Tumorbank)	RRID: N/A
LLC (Lewis Lung Carcinoma)	ATCC	Cat#: CRL-1642; RRID:CVCL_4358
8025 (KPC-derived PDAC)	Derived from KPC (KrasLSL-G12D; Trp53LSL-R172H; Pdx1-Cre) mice (a kind gift of gift from Dieter Saur, TU Munich, Germany)	RRID: N/A
hMSC-Tert adipocytes	This study / as described	RRID: not available
Expi293F	Thermo Fisher Scientific	Cat# A14527; RRID: not available
**Experimental models: Organisms/strains**		
BALB/cAnNCrl mouse	Charles River	Strain code: 028; RRID:IMSR_CRL:028

C57BL/6N mouse	Charles River	Strain code: 027; RRID:IMSR_CRL:027
ApcMin/+ mouse	The Jackson Laboratory	Stock No. 002020; RRID:IMSR_JAX:002020
**Antibodies**		
anti-ADAMTSL4	Santa Cruz Biotechnology	Santa Cruz Biotechnology Cat# sc-134654, RRID:AB_10609105
anti-ATGL (PNPLA2)	Cell Signaling Technology	Cat# 2138; RRID:AB_2167955
anti-VCP	Abcam	Cat# ab111740; RRID:AB_10861709
anti-β-actin	Sigma-Aldrich	Cat# A2228; RRID:AB_476697
anti–phospho-FoxO1 (Thr24)/FoxO3a (Thr32)	Cell Signaling Technology	Cat# 9464; RRID:AB_329842
anti-FoxO3a	Cell Signaling Technology	Cat# 2497; RRID:AB_836876
anti–phospho-STAT3 (Tyr705)	Cell Signaling Technology	Cat# 9145; RRID:AB_2491009
anti-STAT3	Cell Signaling Technology	Cat# 12640; RRID:AB_2629499
anti-LC3B	Cell Signaling Technology	Cat# 3868; RRID:AB_2137707
anti-Bax	Santa Cruz Biotechnology	Cat# sc-20067; RRID:AB_626726
anti-FLAG (DYKDDDDK) tag	Cell Signaling Technology	Cat# 14793; RRID:AB_2572291
anti-HA tag	Cell Signaling Technology	Cell Signaling Technology Cat# 3724, RRID:AB_1549585
anti-Perilipin-1	Cell Signaling Technology	Cat# 3467; RRID:AB_2167270
anti–phospho-SMAD2 (Ser465/Ser467)	Cell Signaling Technology	Cat# 18338; RRID:AB_2798798
anti-SMAD2	Cell Signaling Technology	Cat# 8685; RRID:AB_10889933

anti-Vinculin	Cell Signaling Technology	Cat# 40211; RRID:AB_2728768
anti–6×His tag	Abcam	Cat# ab5000; RRID:AB_304722
anti-V5 tag	Cell Signaling Technology	Cat# 13202; RRID:AB_2687461
mouse anti-rabbit IgG (light chain-specific, D4W3E)	Cell Signaling Technology	Cat# 45262; RRID:AB_2799281
rabbit anti-mouse IgG (light chain-specific, D3V2A), HRP	Cell Signaling Technology	Cat# 58802; RRID:AB_2799549
**Commercial assays**		
Mycoplasma detection PCR kit	Promokine	Cat# PK-CA91-1048; RRID: pending
Free Glycerol Reagent	Sigma-Aldrich	Cat# F6428; RRID: pending
NanoBiT Luciferase Complementation System	Promega	Cat# N2014; RRID: pending
Human ADAMTSL4 ELISA	Cusabio	Cat# CSB-EL001320HU; RRID: pending
Mouse ADAMTSL4 ELISA	Cusabio	Cat# CSB-EL001320MO; RRID: pending
TGF-β1 ELISA	R&D Systems	Cat# DB100C; RRID: pending
IL-6 ELISA	R&D Systems	Cat# D6050; RRID: pending
Activin A ELISA	R&D Systems	Cat# DAC00B; RRID: pending
GDF15 ELISA	R&D Systems	Cat# DGD150; RRID: pending
**Recombinant DNA**		
psPAX2 packaging plasmid	Addgene	Plasmid #12260; RRID:Addgene_12260
pMD2.G envelope plasmid	Addgene	Plasmid #12259; RRID:Addgene_12259
pLV[Exp]-Puro-EF1A>EGFP:T2A:mAdamtsl4	VectorBuilder	RRID: not available (custom vector)
pLV[Exp]-Puro-EF1A>mCherry:T2A:EGFP	VectorBuilder	RRID: not available (custom vector)

MISSION shRNA (Adamtsl4; Tgfb1/LAP; SHC016)	Sigma-Aldrich	TRCN0000080483/84/85; TRCN0000065997; SHC016; RRID: pending
ADAMTSL4-CT-V5-6xHis expression plasmid	Gift (a kind gift of gift from Clair Baldock, Univ. Manchester)	RRID: pending
HA-LAP–TGF-β1 and LAP-FLAG–TGF-β1 constructs	Gifts (a kind gift of gift from Julian Nüchel; Markus Plomann)	RRID: pending
HA tag plasmid	Addgene	Plasmid #55182; RRID:Addgene_55182
FLAG tag plasmid	Addgene	Plasmid #55180; RRID:Addgene_55180
Custom NanoBiT pro–TGF-β1 reporters	BioCat GmbH (custom design)	GBS230125CSB; GBS230125DSB; RRID: not available
Software and algorithms		
GraphPad Prism v10	GraphPad Software	RRID:SCR_002798
Microsoft PowerPoint	Microsoft	RRID: not available
ImageJ/Fiji	NIH / Fiji distribution	RRID:SCR_002285 (Fiji); RRID:SCR_003070 (ImageJ)
BioRender	BioRender.com	RRID:SCR_018361
R (statistical computing environment)	R Foundation	RRID:SCR_001905
clusterProfiler (R package)	Bioconductor	RRID:SCR_016884
**Other**		
LRC-TriCEPS (ligand-receptor capture proteomics)	Dualsystems Biotech AG	TriCEPS Cat# P05201; RRID: not available
EchoMRI body composition analysis	EchoMRI / Echo Medical Systems	RRID: not available
Olink Explore 3072 proteomics platform	Olink	RRID: not available
Beckman Coulter AU480 Chemistry Analyzer	Beckman Coulter	RRID: not available
ChemiDoc MP Imaging System	Bio-Rad	RRID: not available

Q Exactive mass spectrometer	Thermo Fisher Scientific	RRID: not available
TissueLyser II	Qiagen	RRID: not available
QuantStudio 7 Flex Real-Time PCR System	Applied Biosystems	RRID: not available
